# A Genome-Wide DNA Methylation Survey Reveals Salicylic Acid-Induced Distinct Hypomethylation Linked to Defense Responses Against Biotrophic Pathogens

**DOI:** 10.3390/ijms27041935

**Published:** 2026-02-18

**Authors:** Theoni Margaritopoulou, Spyros Foutadakis, Giannis Vatsellas, Martina Samiotaki, Emilia Markellou

**Affiliations:** 1 Laboratory of Mycology, Scientific Directorate of Phytopathology, Benaki Phytopathological Institute, 14561 Kifissia, Greece; 2 4th Department of Internal Medicine, Medical School, National and Kapodistrian University of Athens, 11528 Athens, Greece; 3 Protein Chemistry Facility, Biomedical Sciences Research Center “Alexander Fleming”, 16672 Vari, Greece

**Keywords:** differentially methylated regions, *Podosphaera xanthii*, priming, SCF complex, ubiquitination, whole-genome bisulfite sequencing (WGBS)

## Abstract

DNA methylation is a conserved regulatory mechanism of gene expression, genome stability, and development, and is highly associated with the effective induction of defense responses for plant priming. In the Green Deal era, the use of plant defense inducers (PDIs), compounds that activate defense and prime plants against imminent pathogen attacks, is a safe and environmentally sustainable approach to support plants against pathogens. Though efforts have succeeded at deciphering part of the mode of action of PDIs, more information is needed to understand the underlying pathways of their effectiveness. Here, salicylic acid (SA), loaded in chitosan nanoparticles, increased hypomethylation by more than 25% for 56 genomic regions that corresponded to defense-related genes, such as pectin lyases, defensins and leucine-rich repeat transmembrane protein kinases against the biotrophic fungal pathogen *Podosphaera xanthii*. A genomic region of the promoter of SKP1A, which is a core member of the SCF E3 ubiquitin ligase complex, was found to be a differentially methylated region (DMR), with 60% hypomethylation, both after PDI application and pathogen inoculation, possibly indicating a similar activation mechanism. Examination of this DMR revealed the presence of SA-, auxin-, and defense-related cis-elements. Investigation of the proteins associated with the above cis-elements showed significant upregulation in expression after PDI. Moreover, association of the identified DMR with transcriptomics showed enrichment of the SA pathway. Overall, these findings shed light on the epigenetic mechanisms that underlie SA-related defense priming in plants.

## 1. Introduction

DNA methylation is a highly conserved epigenetic mechanism across eukaryotes controlling numerous biological processes such as imprinting, tissue-specific gene expression, inactivation of transposable elements (TEs), paramutation, and stress responses [1]. DNA methylation involves the insertion of a methyl group at the 5th position of a cytosine residue (5 mC) and is a heritable and fundamental epigenetic modification that can influence the phenotype without altering the DNA nucleotide sequence [2]. In eukaryotes, DNA methylation plays crucial roles in gene regulation and various biological processes that affect plant growth, development and adaptation [2]. While in mammals DNA methylation predominantly occurs in the symmetric CG context and non-CG methylation is prevalent in embryonic stem cells, in plants, DNA methylation occurs at symmetric CG and CHG and asymmetric CHH sites (where H is A, C, or T) [3,4]. The level of DNA methylation notably influences the structure and functionality of the plant genome. A considerable number of molecular mechanisms are affected by DNA methylation, such as transposable element silencing, gene expression regulation and genome stability maintenance, highlighting the importance of this process in the plant cell [5].

A critical outcome of DNA methylation is the regulation of gene expression. Usually, due to promoter methylation, the binding of transcription activators is prevented, leading to the inactivation or reduction of transcription [6,7,8,9]. In addition, the regulation of gene expression can be associated with the integration of different epigenetic mechanisms that influence each other, for example, DNA methylation and histone modifications. The N-terminal tails of histones undergo numerous modifications that are promoted by specific enzymes. Some of these modifications, such as histone acetylation or H3 lysine 4 mono-methylation (H3K4me1), relax the chromatin, thus facilitating transcription [9,10].

Plants, being sessile organisms, must deal with constantly changing environmental conditions in their habitat. For this purpose, plants possess a sophisticated network of response mechanisms that function during exposure to external stimuli. Defense mechanisms are a major part of this network and are activated in response to pathogen attack [11]. This innate immune response operates through conserved signaling mechanisms, such as the recognition of microbe- or damage-associated molecular patterns (MAMPs or DAMPs, respectively), production of reactive oxygen and nitrogen species, and activation of the production of plant defense hormones, such as (SA) and jasmonic acid (JA) [11]. Together, these signaling events lead to a coordinated transcriptional response that regulates the production of defense signals, pathogenesis-related proteins and antimicrobial metabolites [12].

Plants deploy several strategies to defend themselves against pests and pathogens. Key mechanisms are the default response of pattern-triggered immunity (PTI), which responds to microbial molecular patterns, and the evolutionary response of effector-triggered immunity (ETI), which is based on intracellular receptors that recognize immune-suppressive virulence effectors [13]. PTI and ETI are important layers of defense, but plants require additional strategies to survive in hostile environments. A key strategy is the development of acquired or induced resistance, which is forged when plants successfully repel attacks by pests or diseases, including immunological stress memory [14,15,16]. This phenomenon enhances stress resilience and is called “defense priming,” which involves the finely tuned regulation of phytohormone signaling, elevated levels of pattern recognition receptors or dormant defense regulatory elements, and chromatin modifications [17,18].

Defense priming triggers an earlier, faster and/or stronger reaction, leading to enhanced defense response [19]. Regulation of priming, by the timing and amplitude of gene activation, is crucial for the effective induction of defense responses. Evidence shows that priming is controlled by epigenetic changes, such as reduced transposon methylation and trimethylation of lysine 4 of histone subunit H3 (H3K4me3) at defense gene promoters, but it can also be regulated by DNA methylation [20]. In Arabidopsis, mutants with impaired DNA methylation showed increased resistance to hemi-biotrophic pathogens, and infection of Arabidopsis by the hemi-biotrophic pathogen *Pseudomonas syringae* pv. tomato (*Pst*) DC3000 showed reduced DNA methylation [21], revealing the connection between defense response and altered methylation landscape. Additionally, infection of Arabidopsis mutants, with varying levels of immune responses, by *Pst* DC3000, displayed regulation of the expression of *NRPD2* by histone modifications. *NRPD2* is a gene that encodes for the second largest subunit of the plant-specific RNA polymerases IV and V, and this regulation indicates the tight integration of gene expression with methylome changes [22].

Priming of defense responses can be effectively acquired by exogenous application of natural or synthetic compounds, which act as PDIs. There is a plethora of evidence in the literature showing that hormones, such as (SA) and JA; chemical compounds, such as β-aminobutyric acid (BABA), benzothiadiazole (BTH) and acibenzolar-S-methyl (ASM); many volatile organic compounds (VOCs); and chitosan, can effectively stimulate and prime defense responses against various plant pathogens (reviewed in [14,15,19]). The exploration and understanding of the mode of action of these compounds is highly important, as alternative means of control for plant diseases must be developed to alleviate the negative impacts, on humans and the environment, of widespread pesticide use.

The integration of the mode of action of PDIs with a plant’s methylome has gained little attention over the years. Some studies have been performed on SA applications that have shown a positive correlation of methylation changes and gene activation. Treatment of *Vitis amurensis* cell cultures with SA selectively reduced the cytosine DNA methylation of stilbene synthase (STS) genes and stimulated resveratrol production [23], while SA application in tobacco decreased pectin methylation, leading to reduced cell wall Cd accumulation [24]. In this work, we analyzed the DNA methylation response of *Arabidopsis thaliana* plants after inoculation with the biotrophic fungal pathogen *P. xanthii*, the application of SA applied by a chitosan nanoparticle delivery system, and the combination of both treatments. In previous work, we have seen that SA loaded in chitosan nanoparticles (SA-CNPs) can significantly positively regulate both the transcriptome and the proteome in favor of inducing defense against *P. xanthii* and upregulate the SA defense pathway [25]. Here, our results reveal that SA-CNP treatment caused targeted hypomethylation that varied both in methylation context (CpG or CHG) and in intensity with and without pathogen infection. Moreover, integration of the whole-genome bisulfite sequencing (WGBS) results with previous proteomic and transcriptomic data allowed for the functional analysis of genes corresponding to the identified differentially methylated cytosines (DMCs).

This study expands our knowledge of the underlying molecular mechanisms governing defense priming, shows how enhancement of the immune responses in infected plants can be achieved through epigenetic changes, and provides candidates for their exploitation in crop biotechnological strategies.

## 2. Results

### 2.1. SA-CNP Formulation Alters Specific Methylation Contexts in Arabidopsis with and Without Pathogen Inoculation

**Figure 1:**
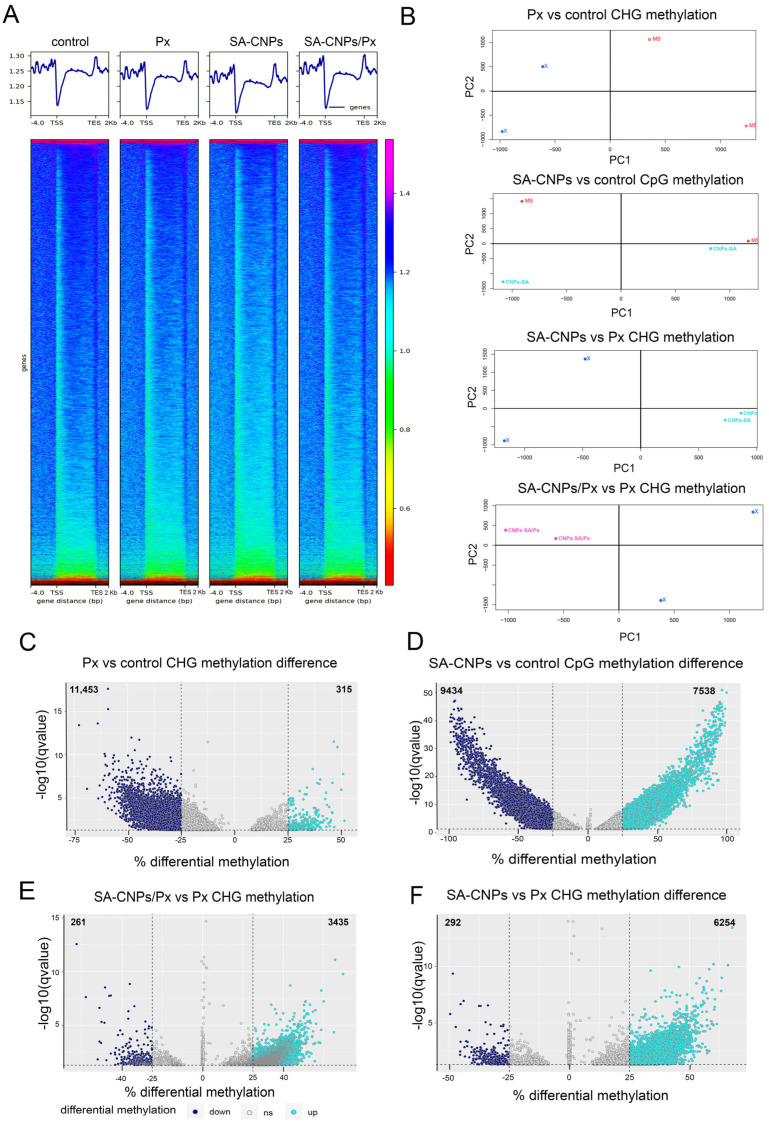
Genome-wide DNA methylation variation in Arabidopsis after pathogen infection and/or SA-CNP application. (**A**) Heatmap of WGBS signals upstream and downstream of the gene body. Scale regions were 4000 bp upstream of the translation starting site (TSS), 2000 bp downstream of the translation end site (TES), and a 5000 bp region on the gene body. Length was plotted using computeMatrix and plotHeatmap tools in deepTools. (**B**) Principal component analysis (PCA) of genome-wide methylation showing separation between the tested treatments in specific methylation (CHG or CpG) contexts. (**C**–**F**) Volcano plots showing the distributions of DMCs in the significantly different treatments. The blue and turquoise dots represent the significantly hypo- and hypermethylated DMCs. Genome-wide significance is determined by false discovery rate (FDR) less than 5% (horizontal dotted line). Vertical dotted lines show >25% hypermethylation and  >25% hypomethylation between treatments.

Previously, we have seen that SA-CNPs can efficiently induce defense responses and prime plants against *P. xanthii* at the very low concentration of 5 ppm [25], while empty CNPs or chitosan showed reduced or similar effects in much higher concentrations. Additionally, these results show that SA-CNPs cause distinct changes both at the transcriptional and at translational levels, when compared with pathogen infection, indicating a targeted mechanism on defense induction. To explore the underlying mechanism associated with gene activation after SA-CNP application, WGBS was performed on Arabidopsis plants treated with SA-CNPs, *P. xanthii* or a combination of both. An average of 23,481,495 million paired-end alignments corresponding to unique best hits were produced (Supplementary Table S1). To characterize global changes in DNA methylation which are indicative of the transcriptional activity status, examination of methylation enrichment around the transcription start site (TSS) was performed using DeepTools (ver. 3.5.6) [26]. The hypomethylation around TSS of Arabidopsis plants following the different treatments showed a relative increase, especially after SA-CNP application, when compared with control conditions, which could indicate transcription activation (Figure 1A). To evaluate methylation profiles between treatments, pair-wise comparisons of interest were performed for each cytosine methylation context (CpG, CHG or CHH). The results show that, in the Px vs. control comparison, only the CHG methylation context differed between treatments, in SA-CNPs vs. control this was the CpG context, in SA-CNPs vs. Px this was the CHG context, and in SA-CNPs/Px vs. Px this was the CHG methylation context (Figure 1B and Figure S2).

To identify significant alterations in DNA methylation between treatments, we performed paired *t*-tests, and cytosines were considered significantly differentially methylated if q (FDR corrected *p*-value) < 0.05 and absolute differential methylation percentage was more than 25%. In the Px vs. control comparison, 11,453 DMCs were hypomethylated and only 325 DMCs were hypermethylated, while in the SA-CNPs vs. control comparison, 9434 DMCs were hypermethylated and 7538 DMCs were hypermethylated, indicating a 23-fold increase in hypermethylation caused by the SA-CNPs, compared with pathogen inoculation (Figure 1C,D). A profound difference between the two treatments was the level of differential cytosine methylation. Pathogen inoculation caused methylation changes up to 60% in hypo- and 35% in hypermethylation, while after SA-CNP application the changes reached 100% in both contexts (Figure 1C,D and Figure S3), indicating high influence of the formulation on the epigenetic landscape of the plants. On the other hand, the SA-CNPs/Px vs. Px and SA-CNPs vs. Px comparisons showed similar hypomethylation in DMCs in both number and percentage level. Particularly, hypomethylated DMCs were 261 and 292 in number, respectively, with a 43% differential methylation level. Additionally, hypermethylated DMCs in these two comparisons were 3435 and 6254, respectively, with a similar 55% differential methylation level (Figure 1E,F and Figure S3). The 1.8-fold increase in the number of hypermethylated DMCs in SA-CNPs vs. Px compared with SA-CNPs/Px vs. Px corroborated the potent effect that the formulation exerted in the plant’s epigenetic background.

### 2.2. Distinct Genomic Regions Have Altered Methylation Imprint After SA-CNP or P. xanthii

**Figure 2:**
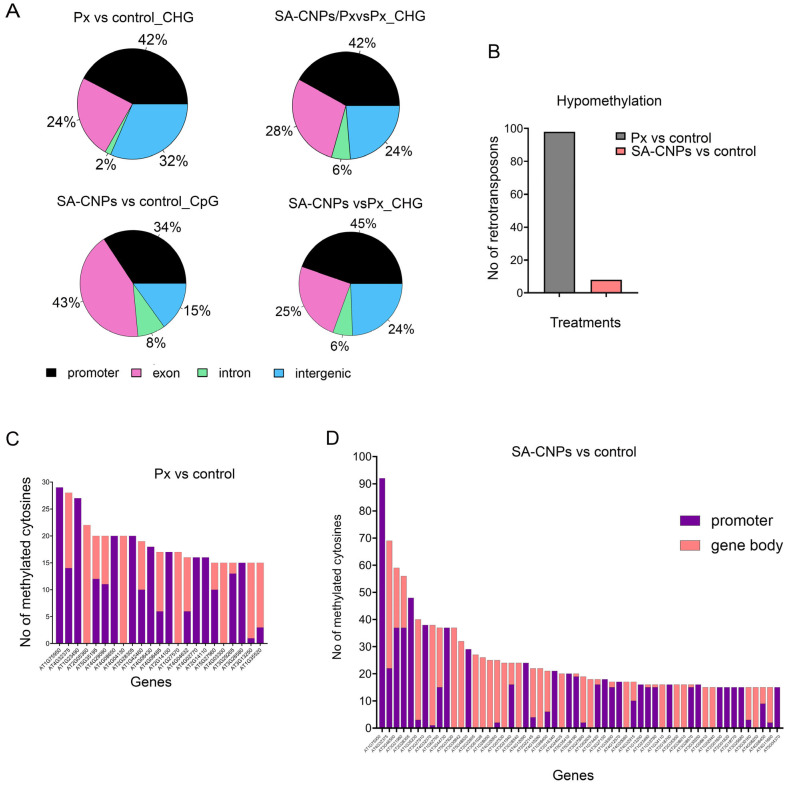
Altered methylation imprints in specific genomic regions between treatments. (**A**) Stratification of differentially methylated cytosines (DMCs) into hypo- and hypermethylated segments representing the promoter, exon, intron or intergenic regions based on their genomic location, indicating the frequency of DMCs per location in the differential significant methylation contexts between treatments. (**B**) Bar plot representing the average number of transposons that were annotated in the hypomethylated segment of the DMCs between Px or SA-CNP treatments compared with control. (**C**,**D**) Enrichment of gene-associated DMCs that are counted in the Px or SA-CNP treatments compared with control. The DMCs are grouped according to their genomic location (promoter or gene body).

Genome-wide distribution analysis of the DMCs showed that each treatment caused both similar and distinct hypomethylation patterns in different genomic regions of the Arabidopsis plants. *P. xanthii* infection-induced hypomethylation changes involved promoters (42%) and intergenic regions (32%), while exons and introns followed with 24% and 2% respectively (Figure 2A). Application of SA-CNPs enhanced hypomethylation in exons reaching 42% and in introns reaching 8%, presenting a 1.75-fold and a 4-fold increase compared with the corresponding values after *P. xanthii* infection, respectively (Figure 2A). Moreover, promoter and intergenic regions containing DMCs were 34% and 15% hypomethylated, respectively, producing decreased 0.2-fold and 0.5-fold differences with the corresponding values after *P. xanthii* infection (Figure 2A). On the other hand, at SA-CNPs/Px vs. Px and SA-CNPs vs. Px comparisons, the regions containing DMCs showed almost identical hypomethylation (Figure 2A). Hypermethylated DMCs were also observed after the treatments in the different genomic regions, following a similar pattern as hypomethylated DMCs (Supplementary Figure S4).

Counting the associated retrotransposons that were identified in DMCs revealed that *P. xanthii* induced cytosine hypomethylation in 99 retrotransposons, while, after SA-CNP application, only 7 were identified (Figure 2B, Supplementary Table S2), demonstrating the targeted action of SA-CNPs on a plant’s epigenome.

### 2.3. SA-CNPs Increase Hypomethylation of Defense-Related Genes Compared with P. xanthii

In-depth analysis of the hypermethylated DMCs did not show any methylation enrichment in regions associated with functional genes after pathogen infection (Supplementary Table S3). On the other hand, SA-CNP application induced cytosine hypermethylation in genomic regions associated with 10 genes (Supplementary Table S3). Among these were *CAX9 (CATION CALCIUM EXCHANGER 3)*, an S-adenosyl-L-methionine-dependent methyltransferases superfamily protein (AT1G31850), *ETL1 (CHROMATIN REMODELING 19)*, and *LCR69*, a defensin-like gene. Hypomethylated DMCs gave more prominent results. Annotation of the functional genes associated with the significant hypomethylated DMCs revealed 56 associated genes with more than 15 DMCs after SA-CNP application and 23 associated genes after pathogen infection (Supplementary Table S4). Among these were many defense-related genes, such as leucine-rich repeat transmembrane protein kinases, pentatricopeptide repeat (PPR) superfamily proteins, pectin-related genes, ubiquitin-related genes and a defensin, which were influenced by SA-CNPs, while *P. xanthii* caused moderate response of a ubiquitin-related gene, a pectin-lyase, two defensins and four Ulp1 proteases (Supplementary Table S4).

The hypomethylated DMCs were present in the promoter, the gene body or both associated identified genes (Figure 2C,D). Notably, the number of DMCs associated with genes was higher in SA-CNPs vs. control, even reaching 92 hypomethylated DMCs in the promoter region of AT1G75950, corresponding to SKP1-like 1A, an E3 ubiquitination ligase protein involved in the SCF E3 ubiquitin ligase complex for subsequent proteasomal degradation of target proteins. The same genomic region showed only 29 hypomethylated DMCs after pathogen infection, indicating a mild effect of *P. xanthii* to the methylated marks on this region, when compared with SA-CNP application (Figure 2C,D, Supplementary Table S4). The second more hypomethylated gene present in both treatments was AT4G32375, a pectin lyase-like superfamily protein with 69 DMCs after SA-CNP application and 28 after *P. xanthii* treatments (Figure 2C,D, Supplementary Table S4).

### 2.4. Hypomethylated DMR That Is Associated with SKP1A Contains Defense-Related Cis-Elements

The search for DMRs, after SA-CNP treatment that would contain the identified DMCs, revealed four hypomethylated DMRs with an mC/totalC ratio of more than 0.2, which are associated with defense responses (Supplementary Table S5). The DMRs were the promoter region of the *SKP1A* gene, the gene body of a defensin-like (AT2G22805), the promoter region of an *SKL2 (SHIKIMATE KINASE LIKE 2)* that enhances resistance under stress conditions, and a disease resistance protein of the TIR-NBS-LRR class (AT4G08450), which acts as an immune receptor against pathogens. Among these, only the corresponding *SKP1A* gene showed prominent differential expression after SA-CNP treatment (Supplementary Figure S5), specifically at the protein level, and this was analyzed further.

**Figure 3:**
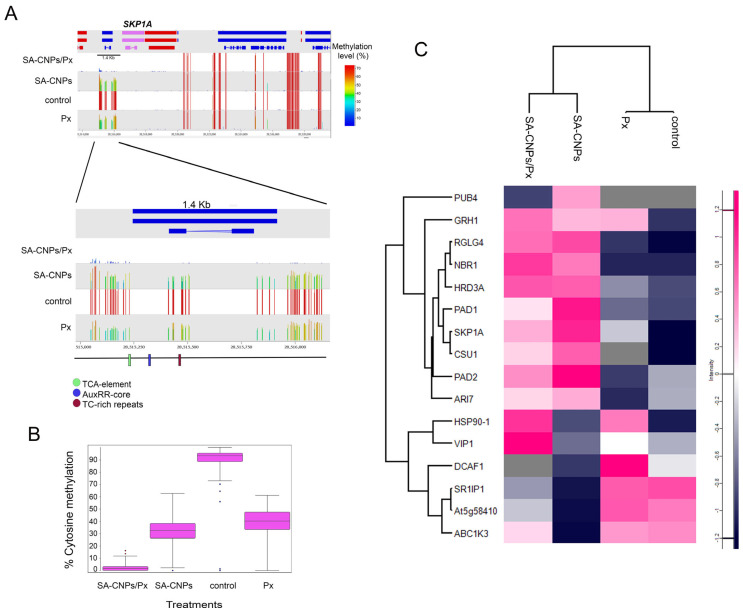
Distribution of DNA methylation and cis-element annotation of the hypomethylated *SKP1A* DMR across treatments and relative protein expression. (**A**) Up: SeqMonk screenshot of a 1.5 Mb region of Arabidopsis chromosome 1 depicting the methylated cytosines. Genes and mRNA are shown in red or blue depending on their direction of transcription (forward and reverse, respectively). The examined *SKP1A* gene is shown in pink. Each color-coded vertical bar in the screenshot represents the methylation value of each methylated cytosine. Down: The zoomed-in view shows methylation over the 1.4 Kb promoter region of the *SKP1A* gene. Each vertical bar represents a single cytosine on the array. The colored boxes on the horizontal black line represent the identified defense-related cis-elements in the selected region. (**B**) Box and whisker plots showing total DNA methylation percentages of the examined *SKP1A* promoter region. The line across the middle of the box shows the median, the upper and lower extremities of the box show the 25th and 75th percentile of the set of data, and the upper and lower black whiskers show the median plus/minus the interquartile (25–75%) range multiplied by 2. Individual points which fall outside this range are shown as filled circles and represent single outlier tiles. (**C**) Heatmap plot, generated with Perseus software (ver. 1.6.15.0), of the indicated expressions of proteins related to E3 ubiquitination. The vertical-colored bar on the right of each plot indicates the value intensity depicting the direction of protein expression as upregulated or downregulated.

Use of SeqMonk (https://www.bioinformatics.babraham.ac.uk/projects/seqmonk/) (accessed on 15 September 2025) for visualization of DNA methylation distribution and examination by PlantCARE (https://bioinformatics.psb.ugent.be/webtools/plantcare/html/) (accessed on 20 November 2025) for cis-elements annotation, revealed the presence of 27 cis-element groups in the 1091 bp genomic region of *SKP1A* (Supplementary Figure S6). Among these, important defense-related cis-elements such as the SA-responsive TCA-element, the auxin-response AuxRR-core-regulatory element, and the TC-rich repeats element, involved in defense and stress responsiveness, were present in the promoter of *SKP1A* (Figure 3A and Figure S6). Quantification of the DNA methylation by SeqMonk showed high methylation under control conditions, significantly reduced methylation after SA-CNP application or *P. xanthii* infection, and even more reduced methylation after combination of both treatments (Figure 3B).

Examination of SKP1A and other E3 ubiquitin ligases in the protein expression profiles from all treatments (control, *P. xanthii*, SA-CNP application, combination of *P. xanthii* and SA-CNP application) showed upregulated expression of 10 out of 16 of the identified proteins (62.5%) after SA-CNP application, including SKP1A (Figure 3C, Supplementary Table S6). Additionally, 8 of the 16 identified proteins (50%) are components of the SCF E3 ubiquitin ligase complex or direct mediators of the complex to the proteasome (Supplementary Table S6).

### 2.5. Upregulation of Proteins Involved with Defense-Related Cis-Elements

**Figure 4:**
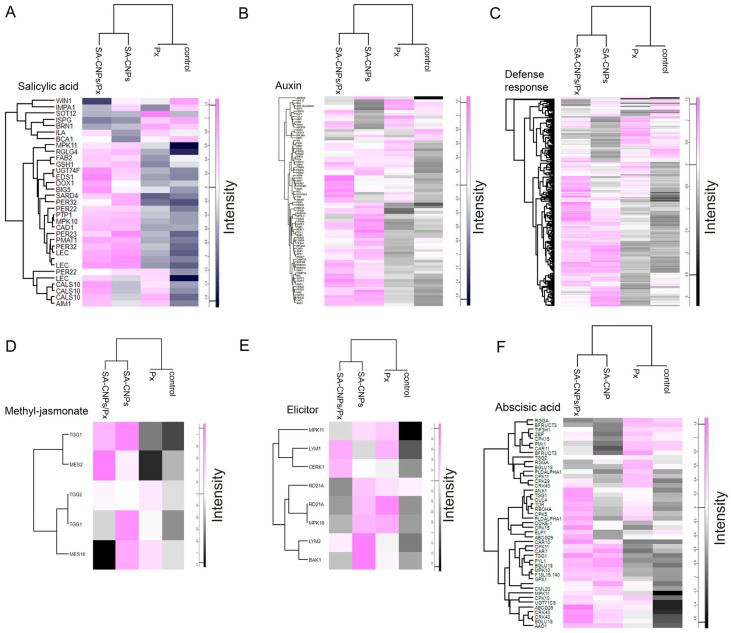
Differential expressions of identified defense-related protein groups between treatments. (**A**–**F**) Heatmap plots, generated with Perseus software (ver. 1.6.15.0), of the indicated expressions of defense-related protein groups. The vertical-colored bar on the right of each plot indicates the value intensity depicting the direction of protein expression as upregulated or downregulated.

Protein expression profiles from all treatments (control, *P. xanthii*, SA-CNP application, combination of *P. xanthii* and SA-CNP application) showed prominent correlation of proteins associated with the above identified cis-regulatory elements in the analyzed DMR and other defense-related protein groups. The examined proteins were associated with functions related to SA, auxin, defense response, methyl jasmonate, elicitors, and abscisic acid (ABA) (Figure 4). Remarkably, almost all protein groups displayed a similar expression pattern between treatments: proteins at control conditions showed the lowest expression levels, followed by *P. xanthii*, the application of SA-CNPs and finally the combination of *P. xanthii* and SA-CNP application showed the higher expression levels (Figure 4A–E). ABA-related proteins also showed similar expression patterns, except from the inverted pattern between *P. xanthii* and SA-CNP application (Figure 4F).

### 2.6. Hypomethylation After SA-CNP Application Is Associated with SA-Related Upregulated Gene Expression

**Figure 5:**
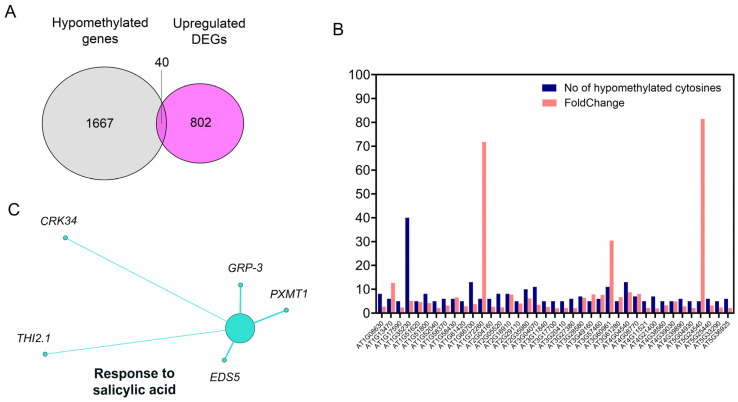
(**A**) Venn diagram displaying shared genes between upregulated differentially expressed genes (DEGs) and enriched hypomethylation in the Arabidopsis SA-CNP-treated samples compared with control. (**B**) Bar plot indicating the number of hypomethylated cytosines and fold change in each of the identified shared genes. (**C**) Illustration depicting the significant enrichment of a functional category identified by ClueGO Cytoscape (ver. 3.10.4) plugin and the corresponding genes.

Integration of the genes corresponding to hypomethylated DMCs, in the SA-CNPs vs. control comparison (>5 DMCs and >25% differential hypomethylation), with the upregulated differentially expressed genes (DEGs) previously identified in this comparison [25] under the same experimental conditions and using three biological triplicates, revealed 40 genes that are common to both gene groups (Figure 5A). Among these was AT1G35230, coding for AGP5 (ARABINOGALACTAN PROTEIN 5) with 40 DMCs and 5.1-fold change (FC), AT4G04540 coding for CRK39 (CYSTEINE-RICH RECEPTOR-LIKE PROTEIN KINASE 39) with 13 DMCs and 8.7 FC, and AT1G51800 coding for IOS1, an LRR receptor-like serine/threonine-protein kinase, with 8 DMCs and 4.2 FC (Figure 5B, Supplementary Table S7). Interestingly, gene ontology (GO) enrichment analysis of the common genes group in Cytoscape (ver. 3.10.4) at medium-to-global network specificity and pathway *p* value ≤ 0.05, showed that *THIONIN2.1* (*THI2.1*), *GLYCINE-RICH PROTEIN 3 (GRP-3)*, *ENHANCED DISEASE SUSCEPTIBILITY 5* (*EDS5*), *1,7-PARAXANTHINE METHYLTRANSFERASE (PXMT1)* and *CYSTEINE-RICH RECEPTOR-LIKE PROTEIN KINASE (CRK34)*, representing 12.5% of the examined genes, enriched the GO term ‘response to salicylic acid’ (Figure 5C).

**Figure 6:**
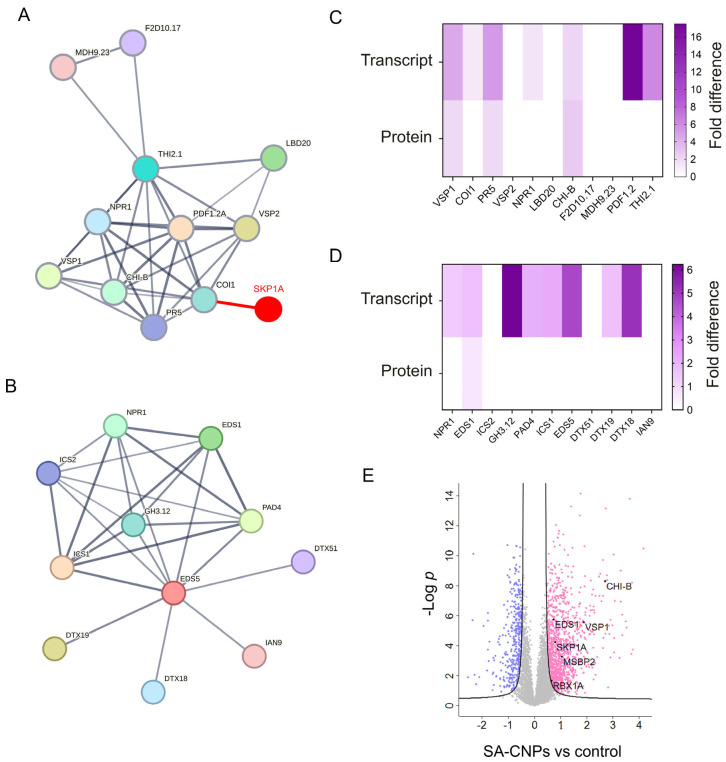
(**A**,**B**) STRING protein–protein interaction (PPI) networks of Arabidopsis THI2.1 (**A**) and EDS5 (**B**) with nine primary associating interactors. Each PPI showed enrichment *p*-value: < 1.0 × 10^−16^. The network nodes are proteins. The edges represent the predicted functional associations; darker edge color represents stronger interaction. The red node and edge in (**A**) show the interaction of SKP1A with COI1 of the network. (**C**,**D**) Heatmap plots, indicating the expressions of the examined genes at transcript and protein level. The vertical-colored bar on the right of each plot indicates the value intensity depicting the direction of expression as upregulated or downregulated. (**E**) Volcano plot, generated with Perseus software (ver 1.6.15.0), of the differentially expressed proteins (DEPs). The log fold change is plotted on the *x*-axis (downregulated proteins with blue color, upregulated with pink) and the negative log10 *p*-value is plotted on the *y*-axis.

Individually subjecting these five genes to functional protein association network analysis by the STRING platform (https://string-db.org/), showed that only THI2.1 and EDS5 could construct a protein–protein interaction network with 10 highly significant primary protein interactors (Figure 6A,B and Figure S7), indicating that their expression is greatly associated with the expression of the interacting proteins. Among these were proteins highly related to SA, such as the key SA immune regulator NPR1, PATHOGENESIS-RELATED PROTEIN 5 (PR5), ENHANCED DISEASE SUSCEPTIBILITY 1 (EDS1) and PHYTOALEXIN DEFICIENT 4 (PAD4). In the same context, searching for interacting proteins of the SKP1A protein that corresponds to the hypomethylated DMR, we found that CORONATINE-INSENSITIVE PROTEIN 1 (COI1) was among the primary 10 interactors, and which is shown to interact with THI2.1 (Figure 6A and Figure S8). Expression analysis of the interacting proteins in both groups at transcriptional and translational levels demonstrated enhanced transcript accumulation to almost all genes between groups and high protein expressions in only VEGETATIVE STORAGE PROTEIN 1 (VSP1), PR5, BASIC ENDOCHITINASE B (CHI-B) and EDS1 (Figure 6C,D). Notably, in this examined SA-CNPs vs. control comparison, SKP1A, along with one of its primary interactors, RING-BOX 1A (RBX1A) (Supplementary Figure S8), showed significantly upregulated protein expression (Figure 6E).

## 3. Discussion

In this study, we aimed to characterize the DNA methylation response to an inducer of resistance and to the biotrophic pathogen *P. xanthii* in *Arabidopsis thaliana* using whole-genome bisulfite sequencing and to associate these findings with proteomic and transcriptomic data. By identifying specific DNA hypomethylation changes in response to the application of SA conjugated to chitosan nanoparticles and demonstrating a connection with gene and protein expression, we provide new insights into the integration of methylome plasticity with the priming of defense responses. Our evaluation of Arabidopsis methylome plasticity upon induction of resistance and pathogen infection allowed for the discovery of distinctive loci participating in the shaping of defense responses. Moreover, we identified significant hypomethylation in the *SKP1A* locus, a member of the SCF E3 ubiquitin ligase complex, to be associated with upregulated SA-related gene and protein expression. Our study highlights the importance of the DNA methylation landscape in defense response mechanisms and its functional implications in induced resistance.

### 3.1. The Arabidopsis Methylome Marks Respond to Treatments

The effect of pathogen infection on DNA methylation has been examined in Arabidopsis and other species, especially for bacterial pathogens, such as the hemi-biotrophic bacterium *P. syringae*, showing that promoters of defense-related genes can be hypomethylated leading to gene activation and resistance upregulation [27,28,29,30]. Studies on fungal pathogens, such as *Fusarium oxysporum* and the necrotrophic fungus *Botrytis cinerea*, have mainly focused on DNA demethylase or RdDM Arabidopsis mutants respectively, showing increased susceptibility to pathogen infection [22,31]. Additionally, there is evidence in citrus plants that DNA methylation changes are associated with defense mechanisms against the gummosis caused by the oomycete *Phytophthora citrophthora* [32]. Our evidence presenting hypomethylation in Arabidopsis after infection by the obligate biotrophic fungal pathogen *P. xanthii* is in accordance with results in wheat progenitor *Aegilops tauschii,* where *Blumeria graminis* f. sp. *tritici* infection also caused hypomethylation in DMRs [33], demonstrating similar response against powdery mildew disease.

PDIs mimic pathogen attack, inducing common defense mechanisms. This indicates that DNA methylation could also be a key mechanism of PDIs in facilitating plants to induce resistance against pathogen infections by altering gene expression. Some PDIs have been identified to enhance plant resistance by altering methylome. In rice, application of 5-azadeoxycytidine, a DNA de-methylating agent, enhances plant resistance to the bacterial pathogen Xanthomonas [34]. Additionally, the chemical SAR inducer β-aminobutyric acid (BABA) has been shown to enhance resistance to various pathogens, including *P. syringae* DC3000, *B. cinerea*, and the oomycete pathogen *Peronospora parasitica*, via its effect on histone modifications and its induction of the expression of defensive genes [35,36,37]. These results are consistent with our findings describing the exploration of the altered methylation landscape by WGBS after the application of SA-CNPs, which function as a PDI. Additionally, they have broadened our knowledge of hypomethylated DMRs and activated genes during defense priming, identifying new constituents of defense priming, such as SKP1A, at the methylation level.

### 3.2. Treatments Generate Specific Modifications of Methylation Marks on Arabidopsis Genomic Regions

Experiments have shown that promoters of defense-related genes may become hypomethylated, which could activate the genes and increase resistance [27,38]. Other examples of characteristic epigenomic alterations can be found after *P. syringae* infection in *Arabidopsis thaliana*, where H3K9 and H3K14 acetylation marks are deposited at promoters of defense-related genes [39,40]. In another work with the same *P. syringae*–*A. thaliana* system, elevated H3K4me3 marks have been found near the promoters of SA-responsive genes, representing a critical mechanism for systemic acquired resistance [41].

Studies on DNA methylation in *Oryza sativa* have shown hypomethylation of a long terminal repeat (LTR) region in the promoter of the rice blast resistance gene, Pit, which may be needed for higher Pit expression against the hemi biotrophic fungal pathogen *Magnaporthe grisea* [42]. Moreover, in another study in *Oryza sativa*, the presence of hypomethylated regions that were predominantly associated with gene promoter regions were observed after infection with the nematode *Meloidogyne graminicola* [43]. Our approach of the estimation of cytosine methylation by WGBS suggests that defense responses to the biotrophic fungal pathogen *P. xanthii* may also be the result of methylation responses in specific genomic regions. Evidence across crop varieties has shown that intergenic regions possess several regulatory regions of defense-responsive genes [44] and could include both junk DNA and functional elements, such as enhancers, the proximal promoter region or the untranslated region (UTR) of a gene [45].

Comparable methylation responses have been observed after the application of flg22, a well-studied peptide corresponding to the most conserved domain of the N-terminus epitope of the bacterial protein flagellin from *Pseudomonas aeruginosa*, which acts as a potent inducer of resistance in plants [43,46]. In this study, similar hypomethylation patterns in promoter, intergenic, exon or intron regions were induced after the application of SA-CNPs, *P. xanthii* infection or the combination of both, highlighting a universal regulatory effect of the methylome on Arabidopsis responses to the treatments. On the other hand, significant differences were observed in the levels of transposon hypomethylation between SA-CNP application and *P. xanthii* infection. This variation could imply that, though the overall hypomethylation patterns between treatments were similar, the SA-CNPs influence specific defense-related pathways compared with the broad stress-related response activated by the pathogen. This notion agrees with previous studies showing that SA-CNPs induce distinct changes in gene expression compared with *P. xanthii* infection [25].

### 3.3. SA-CNP Application Enhances Hypomethylation in Defense-Related Cis Elements Associated with Ubiquitination and Cell Wall Modification Genes

The hypomethylated DMR in SA-CNP samples was the promoter region of *S-Phase Kinase-Associated Protein 1* (*SKP1A*), core subunit of the SCF E3 ubiquitin ligase complex that is formed with CULLIN 1 (CUL1) and RBX1 to ubiquitinate and signal for proteasomal degradation F-box protein targets. The SCF complex plays a critical role in regulating plant immune responses through the ubiquitination pathway by regulating the degradation of negative regulators of plant defense responses [47,48]. There are examples reporting the involvement of the SCF complex in plant immune responses against various pathogens, such as the Tobacco mosaic virus (TMV) [49], *F. oxysporum* [50], *Alternaria brassicicola* [51] and *Plectosphaerella cucumerina* [52]. In these cases, the SCF complex was found to be associated with auxin transport; salicylic or jasmonate signaling; or SAR responses assisting in host defense against the pathogen. In this study, we found that cis elements related to the abovementioned defense pathways were present on the promoter of the *SKP1A* gene, indicating a direct activation mechanism of *SKP1A* for defense. In-depth analysis of the hypomethylation patterns between treatments showed that SA-CNPs increased the level of hypomethylated DMCs and that this increase correlated with the upregulated expression of protein groups corresponding to the identified cis elements, substantiating the connection between defense and *SKP1A* hypomethylation. Moreover, increased expression of the SKP1A protein after SA-CNP application validated the positive regulation of its expression by induced hypomethylation on its promoter. These findings, in combination with the observed RBX1 protein upregulation, could suggest a direct connection of the SCF complex with induced defense mechanisms.

### 3.4. Hypomethylation in DMRs Induced by SA-CNPs Is Linked to the SA Defense Pathway

SA is a multifaceted plant hormone that acts as a key signaling molecule priming plant defenses against biotrophic and hemibiotrophic pathogens. Biotic stresses induce the accumulation of SA in the nucleus as a defense signal [53], and its exogenous application has been observed to significantly enhance plant defense pathways [25,54]. Moreover, plant defense at the methylome and other epigenomic regulatory levels is influenced by SA, as it is shown to influence demethylation in pearl millet [55,56]; increase H3Ac, H4Ac, H3K4me2, and H3K4me3 marks at the PR1 promoter facilitating its expression [57,58]; and generate DNA methylation changes within repetitive sequences and transposons that can regulate neighboring genes in Arabidopsis [28].

Notably, *SKP1A*, the corresponding gene in the hypomethylated DMR after SA-CNP application and component of the SCF (SKP1-CUL1-Fbox) E3 ubiquitin ligase complex, interacts with COI1 to negatively regulate the JA in favor of the SA pathway by ubiquitinating COI1 and regulating its degradation [59]. Therefore, though SKP1A does not function directly within the SA pathway, it influences its activation by competing the JA pathway [60]. At the base of this, one could speculate that the induced SA pathway, after SA-CNP application, activates *SKP1A* by hypomethylating its promoter to negatively regulate components of the JA pathway and antagonizes JA production in the crosstalk of these two hormones for better and enhanced defense responses against biotrophic pathogens. However, further work is needed to determine mechanistically how these pathways integrate under the prism of the methylation landscape and define the roles of the differentially expressed identified proteins. Understanding how PDIs can influence defense responses at the methylation level is essential, as there is evidence showing the significant contribution of methylation to susceptibility or resistance to diseases, indicating a significant role for methylation, one which should be explored under the prism of understanding plant defense activation and enhancement through new, increasingly used, plant protection products.

## 4. Materials and Methods

### 4.1. Plant Material and Growth Conditions

*A. thaliana* Col-0 (wild-type) seeds were obtained from the Arabidopsis Stock Center and used in the experiments. The seeds were sterilized for 20 sec in 70% ethanol, followed by 1.5 min in 20% bleach solution, and rinsed five times with sterile water. The seeds were sown on half-strength Murashige and Skoog (MS) (Duchefa Biochemie B.V, Haarlem, The Netherlands) medium with 1% sucrose and 0.7% agar, stratified at 4 °C in the dark for 2 days, and transferred to a 23 °C growth chamber under long day (16 h light/8 h dark) conditions.

### 4.2. Chitosan Nanoparticles Application

The chitosan nanoparticles loaded with SA used in this study have been previously characterized [25]. *A. thaliana* Col-0 (wild-type) seeds were grown for 5 days in half-strength MS medium Petri dishes and then transferred to half-strength MS medium square plates supplemented with 5 ppm of SA-CNPs.

### 4.3. Pathogen Challenge Accompanied by Hormone Treatment

Pathogen artificial inoculation was performed with a 5 μL high pressure spore suspension (10^7^ spore/mL) of *P. xanthii*. The spore suspension was dropped onto the leaves of 12-day-old Arabidopsis plants grown on half-strength MS medium square plates supplemented with and without 5 ppm SA-CNPs. All inoculated plants were incubated at 23 °C with a 16 h light:8 h dark photoperiod and 65% relative humidity. For WGBS analyses, treated and non-treated 14-day-old plants, with root excision, were collected in liquid nitrogen and stored in −80 °C until use.

### 4.4. BS-Seq Library Construction and Genome Bisulfite Sequencing

Per sample, frozen plant tissue was grounded with liquid nitrogen and genomic DNA was isolated using the SDS procedure of the NucleoSpin Plant II DNA isolation kit (MachereyNagel, Dueren, Germany). A NanoPhotometer^®^ spectrophotometer (IMPLEN, Westlake Village, CA, USA) and agarose gel electrophoresis were used to determine DNA concentration and integrity after the genomic DNA extraction. Next, DNA libraries were prepared for bisulfite sequencing using the NEBNext^®^ Enzymatic Methyl-seq kit (NEB, Ipswich, MA, USA). All sequencing experiments were performed at Biomedical Research Foundation Academy of Athens on an Illumina NovaSeq 6000 sequencing system. Libraries were sequenced with 2 × 150 bp paired end reads at 30× coverage. According to the NIH Roadmap Epigenomics Project the use of two biological replicates with a combined total coverage of 30×, as performed in this study, is sufficient for unbiased genome-wide DNA methylation profiling [61].

### 4.5. Processing of Bisulfite-Treated Reads and Methylation Calling

The raw paired end reads were filtered for low quality reads (Phred score: Q < 30) and adapter using Trimmomatic [62]. The quality filtered reads of each individual sample were aligned to the *Arabidopsis thaliana* reference genome (https://www.ncbi.nlm.nih.gov/search/all/?term=GCF_000001735.4/) (accessed on 15 May 2024) using bowtie 2 (ver. 2.5.4) [63]. The reference genome was first converted in silico into bisulfite converted genome using Bismark tool (ver. 0.22.3) [64] (https://www.bioinformatics.babraham.ac.uk/projects/bismark/, accessed on 15 May 2024). To maintain stringency, only uniquely mapped reads were considered for further analysis. The reads mapping to the same start and end coordinates were considered duplicates and were removed using perl script deduplicate_bismark provided with the Bismark tool (ver. 0.22.3). The mapping file was examined to identify the presence of methylation bias (M-bias). If M-bias was detected, reads were trimmed for biased sequences (~10 bases from 5′ end) to eliminate M-bias. After the mapping of the reads, the absolute methylation profile was obtained using Bismark Methyl Extractor and a whole-genome cytosine report was obtained using the cytosine_report option. A binomial test was executed on the comprehensive genome cytosine report, derived from the Bismark Methyl Extractor, to identify cytosines that were significantly methylated across the entire genome (*Q* < 0.05) for each sample. This analysis considered the computed non-conversion rate and a 5% false-positive sequencing error rate. The mCs represent the absolute profile of methylation per sample. The methylation level of each cytosine was defined as percentage of reads supporting methylation call to total number of reads covering that cytosine. These methylated cytosines were identified as cytosine with a methylation percentage of at least 25% and which were covered by five reads and these were converted to the bedGraph format for visualization. These methylated cytosines were further annotated against the *Arabidopsis thaliana* gene annotation file using BEDTools (ver. 2.28.0) [65]. The detailed pipeline of bisulfite sequencing is shown in Supplementary Figure S1. The summary statistics of methylation data are shown in Supplementary Table S1.

### 4.6. Differential Methylation Analysis

The mapped files generated using Bismark were used to call for differential profiles in terms of differentially methylated cytosines (DMCs). To identify the differential methylation profile between the samples, pair-wise comparison was performed for each cytosine methylation context (CpG, CHG or CHH) using four datasets: pathogen inoculation versus control (XvsC), nanoparticle application versus control (CNPs-SAvsC), pathogen inoculation and nanoparticle application versus pathogen inoculation (CNPs-SA/XvsX), and nanoparticle application versus pathogen inoculation (CNPs-SAvsΧ). The differential methylation annotation and analysis was performed using the R package methylKit [66]. Only those mCs which were covered by a minimum of 5 reads with Q > 30 in each comparison set were considered for analysis. The coverage of each cytosine was normalized using the ‘normalizeCoverage’ option to ensure accuracy in comparisons. The differential methylation was then called using logistic regression controlling for overdispersion (variability among samples). The significance of the differential methylation was assessed using Fisher’s exact test (*p* < 0.05) and corrected for multiple comparisons. Methylation percentage was estimated as 100 * (methylated cytosine/(methylated cytosine + unmethylated cytosine)) for every cytosine in each of the three methylation contexts. The sites that had methylation percentage difference greater than 25% and an FDR < 0.05 were considered as differentially methylated. This is a commonly used strict threshold to ensure both statistical significance and biological importance to comparisons. The DMCs were identified and analyzed separately for each cytosine methylation context. For DMR identification, only regions with a minimum of fifteen (15) cytosines and a frequency (number of methylated cytosines per total cytosines on DMR length) higher than 0.2 were kept for further analysis.

### 4.7. Protein Differential Expression

The protein extraction, digestion and identification have been previously described [25]. Twelve replicates were used for each sample group (4 biological x 3 technical replicates). The results generated were processed statistically and visualized in the Perseus software (1.6.15.0) [67]. Values were log2 transformed, a threshold of 70% of valid values in at least one group was applied and the missing values were replaced from normal distribution. For statistical analysis, Student’s *t*-test was performed, and permutation-based FDR was calculated.

## Data Availability

The raw sequencing data generated in this study have been deposited in the GEO repository under the accession number GSE319280.

## Supplementary Materials

The following supporting information can be downloaded at https://www.mdpi.com/article/10.3390/ijms27041935/s1.
