# Supplementary material for: A Genome-Wide DNA Methylation Survey Reveals Salicylic Acid-Induced Distinct Hypomethylation Linked to Defense Responses Against Biotrophic Pathogens
Source: Int J Mol Sci. 2026 Feb 18;27(4):1935. doi: 10.3390/ijms27041935 (PMC12940366; doi:10.3390/ijms27041935)
Supplement: Supplementary file 1 [file ijms-27-01935-s001.zip › Sup_Table_S1.pdf]

Supplementary Table S1. Summary statistics of methylation mapping. Control: untreated plants, SA-CNPs: plants treated with salicylic acid loaded on chitosan nanoparticles, Px: plants treated with Podosphaera xanthii conidial suspension, SA-CNPs/Px plants with both treatments.

				Methylation context %		
Sample	Total read pairs (RP)	Paired-end alignments with a unique best hit	Mapping efficiency (%)	CG	CHG	CHH
Control_1	45,224,124	26,261,247	58.10	19.6	6.6	1.57
Control_2	43,852,025	24,351,475	55.60	19.8	6.9	1.85
SA-CNPs_1	49,131,536	28,051,962	57.1	19.35	6.4	1.85
SA-CNPs_2	41,077,223	20,293,182	49.5	19	6.3	1.75
Px_1	37,701,050	22,433,919	59.55	18.85	5.9	1.7
Px_2	33,001,268	19,526,778	59.15	19.85	5.7	1.6
SA-CNPs/Px_1	47,760,217	26,913,162	56.4	19.1	6.2	1.65
SA-CNPs/Px_2	40,304,356	20,020,238	49.7	20.1	6.65	1.85
