# Supplementary material for: A Genome-Wide DNA Methylation Survey Reveals Salicylic Acid-Induced Distinct Hypomethylation Linked to Defense Responses Against Biotrophic Pathogens
Source: Int J Mol Sci. 2026 Feb 18;27(4):1935. doi: 10.3390/ijms27041935 (PMC12940366; doi:10.3390/ijms27041935)
Supplement: Supplementary file 1 [file ijms-27-01935-s001.zip › Sup_Table_S2.pdf]

Supplementary table S2. List of identified retrotransposons in the Px vs control and SA-CNPs vs control comparisons.

Px vs control			SA-CNPs vs control		
**Gene**	**No of DMCs**	**Description**	**Gene**	**No of DMCs**	**Description**
AT1G40101	25	gypsy-like retrotransposon family (Athila), has a 5.6e-152 P-value blast match to GB:CAA57397 Athila ORF 1 (Arabidopsis thaliana)	AT2G06400	30	non-LTR retrotransposon family (LINE), has a 1.2e-27 P-value blast match to GB:NP_038603 L1 repeat, Tf subfamily, member 23 (LINE-element) (Mus musculus)
AT5G43800	24	copia-like retrotransposon family, has a 0. P-value blast match to gb|AAG52949| gag/pol polyprotein (Endovir1-1) (Arabidopsis thaliana) (Ty1_Copia-family)	AT1G43940	28	similar to unknown protein [Arabidopsis thaliana] (TAIR:AT3G42540); similar to Retrotransposon gag protein [Medicago truncatula] (GB:ABE81001)
AT5G43415	24	non-LTR retrotransposon family (LINE), has a 6.5e-36 P-value blast match to GB:NP_038605 L1 repeat, Tf subfamily, member 30 (LINE-element) (Mus musculus)	AT1G33817	22	copia-like retrotransposon family, has a 8e-100 P-value blast match to gb|AAO73521| gag-pol polyprotein (Glycine max) (SIRE1) (Ty1_Copia-family)
AT5G07215	24	non-LTR retrotransposon family (LINE), has a 6.9e-34 P-value blast match to GB:NP_038605 L1 repeat, Tf subfamily, member 30 (LINE-element) (Mus musculus)	AT5G35935	21	copia-like retrotransposon family, has a 2.0e-232 P-value blast match to GB:CAA72989 open reading frame 1 (Ty1_Copia-element) (Brassica oleracea)
AT1G36520	24	gypsy-like retrotransposon family (Athila), has a 4.4e-307 P-value blast match to GB:CAA57397 Athila ORF 1 (Arabidopsis thaliana)	AT2G16840	18	copia-like retrotransposon family, has a 7.7e-06 P-value blast match to GB:AAC64917 gag-pol polyprotein (Ty1_Copia-element) (Glycine max)
AT1G35790	24	gypsy-like retrotransposon family (Athila), has a 1.3e-42 P-value blast match to GB:CAA57397 Athila ORF 1 (Arabidopsis thaliana)	AT3G31970	16	gypsy-like retrotransposon family, has a 5.3e-294 P-value blast match to GB:AAD27547 polyprotein (Gypsy_Ty3-element) (Oryza sativa subsp. indica)
AT2G12340	23	non-LTR retrotransposon family (LINE), has a 2.6e-31 P-value blast match to GB:NP_038605 L1 repeat, Tf subfamily, member 30 (LINE-element) (Mus musculus)	AT1G36185	15	copia-like retrotransposon family, has a 0. P-value blast match to GB:CAA72989 open reading frame 1 (Ty1_Copia-element) (Brassica oleracea)
AT1G37669	23	gypsy-like retrotransposon family (Athila), has a 2.0e-258 P-value blast			

		match to GB:CAA57397 Athila ORF 1 (Arabidopsis thaliana)			
AT1G36305	23	gypsy-like retrotransposon family, has a 0. P-value blast match to GB:AAD27547 polyprotein (Gypsy_Ty3-element) (Oryza sativa subsp. indica)			
AT5G34849	22	gypsy-like retrotransposon family, has a 1.5e-166 P-value blast match to GB:AAD27547 polyprotein (Gypsy_Ty3-element) (Oryza sativa subsp. indica)			
AT2G14180	22	gypsy-like retrotransposon family (Athila), has a 3.3e-307 P-value blast match to GB:CAA57397 Athila ORF 1 (Arabidopsis thaliana)			
AT1G41730	22	gypsy-like retrotransposon family (Athila), has a 1.7e-253 P-value blast match to GB:CAA57397 Athila ORF 1 (Arabidopsis thaliana)			
AT5G29577	21	gypsy-like retrotransposon family, has a 2.9e-17 P-value blast match to GB:BAA84458 GAG-POL precursor (gypsy_Ty3-element) (Oryza sativa)gi|5902445|dbj|BAA84458| GAG-POL precursor (Oryza sativa (japonica cultivar-group)) (RIRE2) (Gypsy_Ty3-family)			
AT4G15590	21	non-LTR retrotransposon family (LINE), has a 1.5e-50 P-value blast match to GB:AAA67727 reverse transcriptase (LINE-element) (Mus musculus)			
AT4G05585	21	gypsy-like retrotransposon family, has a 4e-315 P-value blast match to GB:AAD27547 polyprotein (Gypsy_Ty3-element) (Oryza sativa subsp. indica)			
AT3G57586	21	non-LTR retrotransposon family (LINE), has a 7.8e-42 P-value blast match to GB:NP_038605 L1 repeat, Tf subfamily, member 30 (LINE-element) (Mus musculus)			
AT3G44796	21	gypsy-like retrotransposon family, has a 2.2e-307 P-value blast match to GB:AAD27547 polyprotein (Gypsy_Ty3-element) (Oryza sativa subsp. indica)			
AT3G29076	21	gypsy-like retrotransposon family, has a 9e-318 P-value blast match to GB:AAD27547 polyprotein (Gypsy_Ty3-element) (Oryza sativa subsp. indica)			
AT2G14796	21	non-LTR retrotransposon family (LINE), has a 1.7e-05 P-value blast match to GB:NP_038605 L1 repeat, Tf subfamily, member 30 (LINE-element) (Mus musculus)			
AT1G30340	21	copia-like retrotransposon family, has a 0. P-value blast match to GB:CAA72989 open reading frame 1			

		(Ty1_Copia-element) (Brassica oleracea)			
AT5G35113	20	gypsy-like retrotransposon family, has a 2.8e-276 P-value blast match to GB:AAD27547 polyprotein (Gypsy_Ty3-element) (Oryza sativa subsp. indica)			
AT5G27925	20	copia-like retrotransposon family, has a 9.0e-249 P-value blast match to GB:AAC02666 polyprotein (Ty1_Copia-element) (Arabidopsis thaliana)			
AT3G33058	20	gypsy-like retrotransposon family (Athila), has a 2.4e-112 P-value blast match to GB:CAA57397 Athila ORF 1 (Arabidopsis thaliana)			
AT1G43150	20	non-LTR retrotransposon family (LINE), has a 5.3e-25 P-value blast match to GB:NP_038603 L1 repeat, Tf subfamily, member 23 (LINE-element) (Mus musculus)			
AT5G35935	19	copia-like retrotransposon family, has a 2.0e-232 P-value blast match to GB:CAA72989 open reading frame 1 (Ty1_Copia-element) (Brassica oleracea)			
AT2G19840	19	copia-like retrotransposon family, has a 3.5e-301 P-value blast match to GB:CAA31653 polyprotein (Ty1_Copia-element) (Arabidopsis thaliana)			
AT2G13830	19	gypsy-like retrotransposon family, has a 4.4e-283 P-value blast match to GB:AAD22153 polyprotein (Gypsy_Ty3-element) (Sorghum bicolor)			
AT2G01840	19	non-LTR retrotransposon family (LINE), has a 9.6e-34 P-value blast match to GB:NP_038607 L1 repeat, Tf subfamily, member 9 (LINE-element) (Mus musculus)			
AT1G42320	19	gypsy-like retrotransposon family, has a 4.9e-261 P-value blast match to GB:AAD27547 polyprotein (Gypsy_Ty3-element) (Oryza sativa subsp. indica)			
AT5G32345	18	gypsy-like retrotransposon family (Athila), has a 2.2e-183 P-value blast match to GB:CAA57397 Athila ORF 1 (Arabidopsis thaliana)			
AT4G17450	18	copia-like retrotransposon family, has a 1.4e-306 P-value blast match to GB:CAA72989 open reading frame 1 (Ty1_Copia-element) (Brassica oleracea)			
AT4G08078	18	gypsy-like retrotransposon family (Athila), has a 4.6e-276 P-value blast match to GB:CAA57397 Athila ORF 1 (Arabidopsis thaliana)			
AT3G43307	18	gypsy-like retrotransposon family, has a 1.6e-72 P-value blast match to			

		GB:BAA84458 GAG-POL precursor (gypsy_Ty3-element) (Oryza sativa)gi|5902445|dbj|BAA84458| GAG-POL precursor (Oryza sativa (japonica cultivar-group)) (RIRE2) (Gypsy_Ty3-family)			
AT3G31630	18	gypsy-like retrotransposon family, has a 4.6e-319 P-value blast match to GB:AAD27547 polyprotein (Gypsy_Ty3-element) (Oryza sativa subsp. indica)			
AT3G30749	18	gypsy-like retrotransposon family (Athila), has a 9.4e-205 P-value blast match to GB:CAA57397 Athila ORF 1 (Arabidopsis thaliana)			
AT2G14940	18	non-LTR retrotransposon family (LINE), has a 3e-37 P-value blast match to GB:NP_038602 L1 repeat, Tf subfamily, member 18 (LINE-element) (Mus musculus)			
AT2G10250	18	gypsy-like retrotransposon family (Athila), has a 2.0e-157 P-value blast match to GB:CAA57397 Athila ORF 1 (Arabidopsis thaliana)			
AT2G06400	18	non-LTR retrotransposon family (LINE), has a 1.2e-27 P-value blast match to GB:NP_038603 L1 repeat, Tf subfamily, member 23 (LINE-element) (Mus musculus)			
AT1G37735	18	gypsy-like retrotransposon family (Athila), has a 4.9e-189 P-value blast match to GB:CAA57397 Athila ORF 1 (Arabidopsis thaliana)			
AT1G36035	18	copia-like retrotransposon family, has a 0. P-value blast match to gb|AAO73523| gag-pol polyprotein (Glycine max) (SIRE1) (Ty1_Copia-family)			
AT1G31030	18	non-LTR retrotransposon family (LINE), has a 1.0e-41 P-value blast match to GB:NP_038603 L1 repeat, Tf subfamily, member 23 (LINE-element) (Mus musculus)			
AT1G22560	18	non-LTR retrotransposon family (LINE), has a 9.4e-20 P-value blast match to GB:BAA20419 reverse transcriptase (LINE-element) (Mus musculus)			
AT5G28405	17	non-LTR retrotransposon family (LINE), has a 7.9e-30 P-value blast match to GB:AAA67727 reverse transcriptase (LINE-element) (Mus musculus)			
AT4G21360	17	copia-like retrotransposon family, has a 0. P-value blast match to GB:CAA31653 polyprotein (Ty1_Copia-element) (Arabidopsis thaliana)			
AT4G11375	17	copia-like retrotransposon family, has a 5.3e-183 P-value blast match to GB:AAA57005 Hopscotch			

		polyprotein (Ty1_Copia-element) (Zea mays)			
AT4G04050	17	gypsy-like retrotransposon family, has a 2.2e-207 P-value blast match to GB:AAD11615 prpol (gypsy_Ty3-element) (Zea mays)			
AT4G04000	17	non-LTR retrotransposon family (LINE), has a 2.6e-39 P-value blast match to GB:AAB41224 ORF2 (LINE-element) (Rattus norvegicus)			
AT4G03800	17	gypsy-like retrotransposon family, has a 1.5e-125 P-value blast match to GB:AAD19359 polyprotein (gypsy_Ty3-element) (Sorghum bicolor)			
AT4G03795	17	gypsy-like retrotransposon family (Athila), has a 2.2e-111 P-value blast match to GB:CAA57397 Athila ORF 1 (Arabidopsis thaliana)			
AT3G46183	17	copia-like retrotransposon family, has a 2.7e-230 P-value blast match to GB:AAA57005 Hopscotch polyprotein (Ty1_Copia-element) (Zea mays)			
AT3G33069	17	gypsy-like retrotransposon family (Athila), has a 1.6e-294 P-value blast match to GB:CAA57397 Athila ORF 1 (Arabidopsis thaliana)			
AT3G30410	17	copia-like retrotransposon family, has a 5.4e-26 P-value blast match to gb|AAG52950| putative envelope protein (Endovir1-1) (Arabidopsis thaliana) (Ty1_Copia-family)			
AT3G28915	17	non-LTR retrotransposon family (LINE), has a 7.3e-24 P-value blast match to GB:NP_038605 L1 repeat, Tf subfamily, member 30 (LINE-element) (Mus musculus)			
AT2G15870	17	copia-like retrotransposon family, has a 0. P-value blast match to GB:CAA72989 open reading frame 1 (Ty1_Copia-element) (Brassica oleracea)			
AT2G10660	17	gypsy-like retrotransposon family (Athila), has a 4.3e-277 P-value blast match to GB:CAA57397 Athila ORF 1 (Arabidopsis thaliana)			
AT2G10510	17	copia-like retrotransposon family, has a 2.5e-94 P-value blast match to gb|AAG52949| gag/pol polyprotein (Endovir1-1) (Arabidopsis thaliana) (Ty1_Copia-family)			
AT2G05935	17	non-LTR retrotransposon family (LINE), has a 3.9e-28 P-value blast match to GB:NP_038607 L1 repeat, Tf subfamily, member 9 (LINE-element) (Mus musculus)			
AT1G25430	17	non-LTR retrotransposon family (LINE), has a 8.0e-45 P-value blast match to GB:NP_038607 L1 repeat,			

		Tf subfamily, member 9 (LINE-element) (Mus musculus)			
AT1G24640	17	non-LTR retrotransposon family (LINE), has a 4.3e-37 P-value blast match to GB:NP_038602 L1 repeat, Tf subfamily, member 18 (LINE-element) (Mus musculus)			
AT5G33050	16	gypsy-like retrotransposon family (Athila), has a 6.2e-129 P-value blast match to gb|AAL06421|AF378079_1 reverse transcriptase (Athila4) (Arabidopsis thaliana) (Gypsy_Ty3-family)			
AT5G28545	16	non-LTR retrotransposon family (LINE), has a 2.7e-08 P-value blast match to GB:AAB41224 ORF2 (LINE-element) (Rattus norvegicus)			
AT5G24915	16	non-LTR retrotransposon family (LINE), has a 3e-38 P-value blast match to GB:NP_038603 L1 repeat, Tf subfamily, member 23 (LINE-element) (Mus musculus)			
AT4G08275	16	gypsy-like retrotransposon family (Athila), has a 3.3e-99 P-value blast match to GB:CAA57397 Athila ORF 1 (Arabidopsis thaliana)			
AT4G05556	16	gypsy-like retrotransposon family (Athila), has a 1.2e-223 P-value blast match to GB:CAA57397 Athila ORF 1 (Arabidopsis thaliana)			
AT3G44605	16	non-LTR retrotransposon family (LINE), has a 9e-38 P-value blast match to GB:AAB41224 ORF2 (LINE-element) (Rattus norvegicus)			
AT3G42715	16	gypsy-like retrotransposon family (Athila), has a 4.4e-209 P-value blast match to GB:CAA57397 Athila ORF 1 (Arabidopsis thaliana)			
AT3G42313	16	gypsy-like retrotransposon family, has a 7.2e-227 P-value blast match to GB:AAD27547 polyprotein (Gypsy_Ty3-element) (Oryza sativa subsp. indica)			
AT3G30708	16	gypsy-like retrotransposon family, has a 4.3e-90 P-value blast match to gb|AAL06420|AF378078_1 reverse transcriptase (Arabidopsis thaliana) (Gypsy_Ty3-family)			
AT2G12760	16	gypsy-like retrotransposon family, has a 5.3e-303 P-value blast match to GB:AAD27547 polyprotein (Gypsy_Ty3-element) (Oryza sativa subsp. indica)			
AT2G09870	16	gypsy-like retrotransposon family (Athila), has a 5.6e-224 P-value blast match to GB:CAA57397 Athila ORF 1 (Arabidopsis thaliana)			
AT1G70010	16	copia-like retrotransposon family, has a 3.2e-289 P-value blast match to GB:CAA72989 open reading frame 1			

		(Ty1_Copia-element) (Brassica oleracea)			
AT1G36720	16	gypsy-like retrotransposon family, has a 2.3e-308 P-value blast match to GB:AAD27547 polyprotein (Gypsy_Ty3-element) (Oryza sativa subsp. indica)			
AT5G38705	15	gypsy-like retrotransposon family, has a 4.4e-305 P-value blast match to GB:AAD27547 polyprotein (Gypsy_Ty3-element) (Oryza sativa subsp. indica)			
AT5G35535	15	non-LTR retrotransposon family (LINE), has a 2.2e-31 P-value blast match to GB:NP_038604 L1 repeat, Tf subfamily, member 26 (LINE-element) (Mus musculus)			
AT5G29032	15	gypsy-like retrotransposon family (Athila), has a 2e-191 P-value blast match to GB:CAA57397 Athila ORF 1 (Arabidopsis thaliana)			
AT5G26236	15	gypsy-like retrotransposon family, has a 3.5e-307 P-value blast match to GB:AAD27547 polyprotein (Gypsy_Ty3-element) (Oryza sativa subsp. indica)			
AT4G08138	15	gypsy-like retrotransposon family, has a 5.7e-299 P-value blast match to GB:AAD27547 polyprotein (Gypsy_Ty3-element) (Oryza sativa subsp. indica)			
AT4G07830	15	gypsy-like retrotransposon family, has a 6.3e-159 P-value blast match to GB:CAA73042 polyprotein (Gypsy_Ty3-element) (Ananas comosus)			
AT4G06684	15	copia-like retrotransposon family, has a 4.2e-22 P-value blast match to gb|AAG52950| putative envelope protein (Endovir1-1) (Arabidopsis thaliana) (Ty1_Copia-family)			
AT4G06506	15	gypsy-like retrotransposon family (Athila), has a 3e-255 P-value blast match to GB:CAA57397 Athila ORF 1 (Arabidopsis thaliana)			
AT4G05073	15	copia-like retrotransposon family, has a 5e-300 P-value blast match to GB:CAA31653 polyprotein (Ty1_Copia-element) (Arabidopsis thaliana)			
AT4G04550	15	copia-like retrotransposon family, has a 1.9e-10 P-value blast match to gb|AAG52950| putative envelope protein (Endovir1-1) (Arabidopsis thaliana) (Ty1_Copia-family)			
AT3G43304	15	gypsy-like retrotransposon family (Athila), has a 5e-231 P-value blast match to GB:CAA57397 Athila ORF 1 (Arabidopsis thaliana)			
AT3G42545	15	non-LTR retrotransposon family (LINE), has a 5.5e-26 P-value blast			

		match to GB:NP_038605 L1 repeat, Tf subfamily, member 30 (LINE-element) (Mus musculus)			
AT3G42431	15	gypsy-like retrotransposon family (Athila), has a 1.5e-78 P-value blast match to GB:CAA57397 Athila ORF 1 (Arabidopsis thaliana)			
AT3G33084	15	gypsy-like retrotransposon family, has a 1.6e-186 P-value blast match to GB:AAD22153 polyprotein (Gypsy_Ty3-element) (Sorghum bicolor)			
AT3G30213	15	gypsy-like retrotransposon family (Athila), has a 3.2e-53 P-value blast match to GB:CAA57397 Athila ORF 1 (Arabidopsis thaliana)			
AT3G28153	15	non-LTR retrotransposon family (LINE), has a 1.3e-30 P-value blast match to GB:AAB41224 ORF2 (LINE-element) (Rattus norvegicus)			
AT3G26614	15	non-LTR retrotransposon family (LINE), has a 3.0e-39 P-value blast match to GB:NP_038607 L1 repeat, Tf subfamily, member 9 (LINE-element) (Mus musculus)			
AT2G14190	15	gypsy-like retrotransposon family (Athila), has a 8.5e-177 P-value blast match to GB:CAA57397 Athila ORF 1 (Arabidopsis thaliana)			
AT2G13490	15	non-LTR retrotransposon family (LINE), has a 3.3e-19 P-value blast match to GB:NP_038607 L1 repeat, Tf subfamily, member 9 (LINE-element) (Mus musculus)			
AT2G11450	15	gypsy-like retrotransposon family (Athila), has a 1.7e-208 P-value blast match to GB:CAA57397 Athila ORF 1 (Arabidopsis thaliana)			
AT2G04670	15	gypsy-like retrotransposon family, has a 1.2e-313 P-value blast match to GB:AAD27547 polyprotein (Gypsy_Ty3-element) (Oryza sativa subsp. indica)			
AT1G43715	15	copia-like retrotransposon family, has a 2.6e-130 P-value blast match to GB:BAA78424 polyprotein (Ty1_Copia-element) (Arabidopsis thaliana)gi|4996363|dbj|BAA78424| polyprotein (AtRE2) (Arabidopsis thaliana) (Ty1_Copia-element)			
AT1G43444	15	gypsy-like retrotransposon family, has a 7.2e-242 P-value blast match to GB:AAD27547 polyprotein (Gypsy_Ty3-element) (Oryza sativa subsp. indica)			
AT1G42924	15	copia-like retrotransposon family, has a 0. P-value blast match to gb|AAG52949| gag/pol polyprotein (Endovir1-1) (Arabidopsis thaliana) (Ty1_Copia-family)			

AT1G36300	15	gypsy-like retrotransposon family (Athila), has a 1e-218 P-value blast match to GB:CAA57397 Athila ORF 1 (Arabidopsis thaliana)			
AT1G36185	15	copia-like retrotransposon family, has a 0. P-value blast match to GB:CAA72989 open reading frame 1 (Ty1_Copia-element) (Brassica oleracea)			
