# Supplementary material for: A Genome-Wide DNA Methylation Survey Reveals Salicylic Acid-Induced Distinct Hypomethylation Linked to Defense Responses Against Biotrophic Pathogens
Source: Int J Mol Sci. 2026 Feb 18;27(4):1935. doi: 10.3390/ijms27041935 (PMC12940366; doi:10.3390/ijms27041935)
Supplement: Supplementary file 1 [file ijms-27-01935-s001.zip › Sup_Table_S3.pdf]

Supplementary Tabe S3. Annotation of the functional genes associated with hypermethylated DMCs.

SA-CNPs vs control				Px vs control			
**Locus**	**Description**	**Function**	**No of hypo-DMCs**	**Locus**	**Description**	**Function**	**No of hypo-DMCs**
AT3G14070	cation exchanger 9 (CAX9)		41				
AT1G31850	S-adenosyl-L-methionine-dependent methyltransferases superfamily protein		36				
AT2G02090	ETL1	helicase activity, DNA binding, ATP binding, nucleic acid binding	30				
AT2G02100	low-molecular-weight cysteine-rich 69 (LCR69)	peptidase inhibitor activity	30				
AT1G31870	unknown protein	function unknown	20				
AT1G32180	cellulose synthase-like D6 (CSLD6)	cellulose synthase activity, transferase activity, transferring glycosyl groups	19				
AT4G04890	protodermal factor 2 (PDF2)	DNA binding, sequence-specific DNA binding transcription factor activity	17				
AT1G20925	Auxin efflux carrier family protein	auxin:hydrogen symporter activity	15				
AT1G31860	AT-IE	phosphoribosyl-AMP cyclohydrolase activity, phosphoribosyl-ATP diphosphatase activity	15				
AT3G03060	P-loop containing nucleoside triphosphate hydrolases superfamily protein	ATPase activity, zinc ion binding	15				
