# Supplementary material for: A Genome-Wide DNA Methylation Survey Reveals Salicylic Acid-Induced Distinct Hypomethylation Linked to Defense Responses Against Biotrophic Pathogens
Source: Int J Mol Sci. 2026 Feb 18;27(4):1935. doi: 10.3390/ijms27041935 (PMC12940366; doi:10.3390/ijms27041935)
Supplement: Supplementary file 1 [file ijms-27-01935-s001.zip › Sup_Table_S4.pdf]

Supplementary Tabe S4. Annotation of the functional genes associated with significant hypomethylated DMCs.

SA-CNPs vs control				Px vs control			
**Locus**	**Description**	**Function**	**No of hypo-DMCs**	**Locus**	**Description**	**Function**	**No of hypo-DMCs**
AT1G75950	S phase kinase-associated protein 1 (SKP1)	ubiquitin-protein ligase activity, protein binding	92	AT1G75950	S phase kinase-associated protein 1 (SKP1)	ubiquitin-protein ligase activity, protein binding	29
AT4G32375	Pectin lyase-like superfamily protein	polygalacturonase activity	69	AT4G32375	Pectin lyase-like superfamily protein	polygalacturonase activity	28
AT3G46500	2-oxoglutarate (2OG) and Fe(II)-dependent oxygenase superfamily protein	oxidoreductase activity, iron ion binding	59	AT1G23490	ADP-ribosylation factor 1 (ARF1)	protein binding, phospholipase activator activity, GTP binding	27
AT3G21980	Domain of unknown function (DUF26)	function unknown	56	AT2G05360	F-box associated ubiquitination effector family protein		22
AT2G28305	LONELY GUY 1 (LOG1)		48	AT5G35195	Defensin-like (DEFL) family protein		20
AT1G35230	Arabinogalactan protein 5 (AGP5)		40	AT4G29090	Ribonuclease H-like superfamily protein	RNA binding, RNA-directed DNA polymerase activity, nucleic acid binding	20
AT3G27910	Galactose oxidase/kelch repeat superfamily protein	function unknown	38	AT4G08650	Hypothetical protein		20
AT4G32370	Pectin lyase-like superfamily protein	polygalacturonase activity	38	AT4G04130	Similar to Ulp1 protease family protein		20
AT1G62760	Plant invertase/pectin methylesterase inhibitor superfamily protein	enzyme inhibitor activity, pectinesterase inhibitor activity, pectinesterase activity	37	AT2G28305	LONELY GUY 1 (LOG1)		20
AT3G44730	Kinesin-like protein 1 (KP1)	microtubule motor activity, ATP binding	37	AT1G42460	Similar to Ulp1 protease family protein		19
AT5G37620	Cysteine/Histidine-rich C1 domain family protein	zinc ion binding	37	AT4G08430	Ulp1 protease family protein	cysteine-type peptidase activity	18
AT3G30842	Pleiotropic drug resistance 10 (PDR10)	nucleoside-triphosphatase activity, ATPase activity, nucleotide binding, ATP binding	32	AT4G08485	Defensin-like (DEFL) family protein		17

AT5G48820	Inhibitor/interactor with cyclin-dependent kinase (ICK6)		29	AT2G14100	Pytochrome P450, family 705, subfamily A, polypeptide 13 (CYP705A13)	electron carrier activity, monooxygenase activity, iron ion binding, oxygen binding, heme binding	17
AT2G22805	Defensin-like (DEFL) family protein		27	AT1G27570	Phosphatidylinositol 3- and 4-kinase family protein	inositol or phosphatidylinositol kinase activity, binding, phosphotransferase activity, alcohol group as acceptor	17
AT3G61028	Putative endonuclease or glycosyl hydrolase	function unknown	26	AT4G04632	Protein kinase superfamily protein	protein kinase activity, ATP binding	16
AT1G28000	Pentatricopeptide repeat (PPR) superfamily protein	function unknown	25	AT4G02770	Photosystem I subunit D-1 (PSAD-1)	molecular_function unknown	16
AT4G22060	F-box domain, cyclin-like (DUF295)	function unknown	25	AT2G14110	Haloacid dehalogenase-like hydrolase (HAD) superfamily protein		16
AT1G07530	SCARECROW-like 14 (SCL14)	function unknown	24	AT5G27960	AGAMOUS-like 90 (AGL90	DNA binding, sequence-specific DNA binding transcription factor activity	15
AT3G21940	Receptor protein kinase-related	function unknown	24	AT4G03300	Similar to Ulp1 protease family protein		15
AT3G28540	P-loop containing nucleoside triphosphate hydrolases superfamily protein	nucleoside-triphosphatase activity, ATPase activity, nucleotide binding, ATP binding	24	AT3G29265	Similar to zinc knuckle (CCHC-type) family protein		15
AT4G12090	Cornichon family protein	function unknown	24	AT3G28580	P-loop containing nucleoside triphosphate hydrolases superfamily protein	nucleoside-triphosphatase activity, ATPase activity, nucleotide binding, ATP binding	15
AT2G22145	ECA1 gametogenesis related family protein	function unknown	22	AT3G13250	Similar to AT hook motif-containing protein-related		15
AT4G10290	RmlC-like cupins superfamily protein	Protein of unknown function DUF861	22	AT1G35520	Auxin response factor 15 (ARF15)	sequence-specific DNA binding transcription factor activity	15
AT1G68400	Leucine-rich repeat transmembrane protein kinase family protein	protein serine/threonine kinase activity, kinase activity, ATP binding	21				
AT2G16340	unknown protein		21				
AT4G24025	tRNA-Gly (anticodon: CCC)		20				

AT5G35410	SALT OVERLY SENSITIVE 2 (SOS2)		20				
AT5G38190	Myosin heavy chain-like protein		20				
AT3G27800	Unknown protein		19				
AT1G62935	Unknown protein		18				
AT1G74420	Fucosyltransferase 3 (FUT3)	transferase activity, transferring glycosyl groups, fucosyltransferase activity	18				
AT3G27150	Galactose oxidase/kelch repeat superfamily protein		18				
AT1G35900	Unknown protein		17				
AT4G12570	Ubiquitin protein ligase 5 (UPL5)		17				
AT4G20080	Calcium-dependent lipid-binding (CaLB domain) plant phosphoribosyltransferase family protein		17				
AT4G32915			17				
AT1G13200	F-box and associated interaction domains-containing protein		16				
AT1G23490	ADP-ribosylation factor 1 (ARF1)	protein binding, phospholipase activator activity, GTP binding	16				
AT1G33780	Protein of unknown function (DUF179)		16				
AT1G34110	Leucine-rich receptor-like protein kinase family protein	protein serine/threonine kinase activity, protein kinase activity, ATP binding	16				
AT2G18700	Trehalose phosphatase/synthase 11 (TPS11)	transferase activity, transferring glycosyl groups	16				
AT2G35500	Shikimate kinase like 2 (SKL2)	shikimate kinase activity, ATP binding	16				
AT2G38010	Neutral/alkaline non-lysosomal ceramidase	ceramidase activity	16				

AT3G28670	Oxidoreductase, zinc-binding dehydrogenase family protein	oxidoreductase activity, zinc ion binding	16				
AT3G30250	Unknown protein		16				
AT1G08610	Pentatricopeptide repeat (PPR) superfamily protein		15				
AT1G52940	purple acid phosphatase 5 (PAP5)		15				
AT2G01650	Plant UBX domain-containing protein 2 (PUX2)		15				
AT2G01820	Leucine-rich repeat protein kinase family protein	protein serine/threonine kinase activity, protein kinase activity, ATP binding	15				
AT3G19770	VPS9A	Rho guanyl-nucleotide exchange factor activity	15				
AT3G25680	Unknown protein		15				
AT3G30160	Unknown protein		15				
AT4G08250	GRAS family transcription factor		15				
AT4G08450	Disease resistance protein (TIR-NBS-LRR class) family	transmembrane receptor activity, nucleoside-triphosphatase activity, nucleotide binding, ATP binding	15				
AT4G11660	AT-HSFB2B	transcription repressor activity, sequence-specific DNA binding transcription factor activity	15				
AT5G04370	S-adenosyl-L-methionine-dependent methyltransferases superfamily protein (NAMT1)	methylates nicotinic acid to yield methyl nicotinate	15				
