# Supplementary material for: A Genome-Wide DNA Methylation Survey Reveals Salicylic Acid-Induced Distinct Hypomethylation Linked to Defense Responses Against Biotrophic Pathogens
Source: Int J Mol Sci. 2026 Feb 18;27(4):1935. doi: 10.3390/ijms27041935 (PMC12940366; doi:10.3390/ijms27041935)
Supplement: Supplementary file 1 [file ijms-27-01935-s001.zip › Sup_Table_S5.pdf]

Supplementary Table S5. List of the methylated regions, that contain the identified hypomethylated DMCs. The annotated differentially methylated regions (DMRs) show a mC / total C ratio more than 0.2.

Locus ID	Description	No of hypo-DMCs	Genomic region's length	Total C	Hypo mC/total C ratio	comments
AT1G75950	S phase kinase-associated protein 1 (SKP1); FUNCTIONS IN: ubiquitin-protein ligase activity, protein binding; INVOLVED IN: negative regulation of DNA recombination, response to cadmium ion, mitosis, male meiosis, ubiquitin-dependent protein catabolic process	92	1091	400	0.23	
AT4G32375	Pectin lyase-like superfamily protein; FUNCTIONS IN: polygalacturonase activity; INVOLVED IN: carbohydrate metabolic process	69	5088	1546	0.04	
AT3G46500	2-oxoglutarate (2OG) and Fe(II)-dependent oxygenase superfamily protein; FUNCTIONS IN: oxidoreductase activity, iron ion binding; INVOLVED IN: oxidation reduction	59	4564	1592	0.013	
AT3G21980	Domain of unknown function (DUF26); FUNCTIONS IN: molecular_function unknown; INVOLVED IN: biological_process unknown	56	2312	808	0.07	
AT2G28305	LONELY GUY 1 (LOG1); CONTAINS InterPro DOMAIN/s: Conserved hypothetical protein CHP00730 (InterPro:IPR005269); BEST Arabidopsis thaliana protein match is: lysine decarboxylase family protein (TAIR:AT2G37210)	48				not detected
AT1G35230	arabinogalactan protein 5 (AGP5); BEST Arabidopsis thaliana protein match is: arabinogalactan protein 10 (TAIR:AT4G09030)	40	2231	757	0.05	
AT3G27910	Galactose oxidase/kelch repeat superfamily protein; FUNCTIONS IN: molecular_function unknown; INVOLVED IN: biological_process unknown; LOCATED IN: endomembrane system	38	2766	836	0.045	
AT4G32370	Pectin lyase-like superfamily protein; FUNCTIONS IN: polygalacturonase activity; INVOLVED IN: carbohydrate metabolic process	38				same region as in AT4G32375
AT1G62760	Plant invertase/pectin methylesterase inhibitor superfamily protein; FUNCTIONS IN: enzyme inhibitor activity, pectinesterase inhibitor activity, pectinesterase activity	37	1498	555	0.07	
AT3G44730	kinesin-like protein 1 (KP1); FUNCTIONS IN: microtubule motor activity, ATP binding; INVOLVED IN: microtubule-based movement	37				
AT5G37620	Cysteine/Histidine-rich C1 domain family protein; FUNCTIONS IN: zinc ion binding; INVOLVED IN: biological_process unknown	37				
AT3G30842	pleiotropic drug resistance 10 (PDR10); FUNCTIONS IN: nucleoside-triphosphatase activity, ATPase activity, nucleotide binding, ATP binding; INVOLVED IN: drug transmembrane transport	32	1613	839	0.044	

AT5G48820	inhibitor/interactor with cyclin-dependent kinase (ICK6); CONTAINS InterPro DOMAIN/s: Cyclin-dependent kinase inhibitor (InterPro:IPR003175)	29				
AT2G22805	Defensin-like (DEFL) family protein; LOCATED IN: endomembrane system; BEST Arabidopsis thaliana protein match is: Defensin-like (DEFL) family protein (TAIR:AT2G22807)	27	301	117	0.23	
AT3G61028	Putative endonuclease or glycosyl hydrolase; FUNCTIONS IN: molecular_function unknown; INVOLVED IN: biological_process unknown	26	415	171	0.15	
AT1G28000	Pentatricopeptide repeat (PPR) superfamily protein; FUNCTIONS IN: molecular_function unknown; INVOLVED IN: biological_process unknown	25	584	260	0.1	
AT4G22060	CONTAINS InterPro DOMAIN/s: F-box domain, cyclin-like (InterPro:IPR001810), Protein of unknown function DUF295 (InterPro:IPR005174)	25				
AT1G07530	SCARECROW-like 14 (SCL14); CONTAINS InterPro DOMAIN/s: Transcription factor GRAS (InterPro:IPR005202); BEST Arabidopsis thaliana protein match is: GRAS family transcription factor (TAIR:AT2G29060)	24				
AT3G21940	Receptor protein kinase-related; FUNCTIONS IN: molecular_function unknown; INVOLVED IN: biological_process unknown	24	1090	398	0.06	
AT3G28540	P-loop containing nucleoside triphosphate hydrolases superfamily protein; FUNCTIONS IN: nucleoside-triphosphatase activity, ATPase activity, nucleotide binding, ATP binding	24	1138	477	0.05	
AT4G12090	Cornichon family protein; FUNCTIONS IN: molecular_function unknown; INVOLVED IN: intracellular signaling pathway	24				
AT2G22145	ECA1 gametogenesis related family protein; CONTAINS InterPro DOMAIN/s: Protein of unknown function DUF1278 (InterPro:IPR010701)	22				
AT4G10290	RmlC-like cupins superfamily protein; CONTAINS InterPro DOMAIN/s: Protein of unknown function DUF861, cupin-3 (InterPro:IPR008579), Cupin, RmlC-type (InterPro: IPR011051)	22				
AT1G68400	leucine-rich repeat transmembrane protein kinase family protein; FUNCTIONS IN: protein serine/threonine kinase activity, kinase activity, ATP binding; INVOLVED IN: transmembrane receptor protein tyrosine kinase signaling pathway, protein amino acid phosphorylation	21	531	271	0.08	
AT2G16340	unknown protein	21				
AT4G24025	tRNA-Gly (anticodon: CCC)	20				
AT5G35410	SALT OVERLY SENSITIVE 2 (SOS2); CONTAINS InterPro DOMAIN/s: Protein kinase, ATP binding site (InterPro:IPR017441), Serine/threonine-protein kinase domain (InterPro:IPR002290), NAF/FISL domain (InterPro:IPR018451), Serine/threonine-protein kinase-like domain (InterPro:IPR017442), Protein kinase-like domain (InterPro:IPR011009), Serine/threonine-protein kinase, active site (InterPro:IPR008271), NAF domain (InterPro:IPR004041), CBL-interacting protein kinase (InterPro:IPR020660), Protein kinase, catalytic domain (InterPro:IPR000719),	20				

	Calcium/calmodulin-dependent protein kinase-like (InterPro:IPR020636)					
AT5G38190	INVOLVED IN: biological_process unknown; LOCATED IN: chloroplast; BEST Arabidopsis thaliana protein match is: myosin heavy chain-related (TAIR:AT1G32010)	20				
AT3G27800	unknown protein	19				
AT1G62935	unknown protein	18				
AT1G74420	fucosyltransferase 3 (FUT3); FUNCTIONS IN: transferase activity, transferring glycosyl groups, fucosyltransferase activity; INVOLVED IN: cell wall biogenesis	18				
AT3G27150	Galactose oxidase/kelch repeat superfamily protein; CONTAINS InterPro DOMAIN/s: Galactose oxidase/kelch, beta-propeller (InterPro:IPR011043), Kelch repeat type 1 (InterPro:IPR006652), Kelch related (InterPro:IPR013089), Kelch-type beta propeller (InterPro:IPR015915)	18	1623	377	0.05	
AT1G35900	unknown protein	17				
AT4G12570	ubiquitin protein ligase 5 (UPL5); CONTAINS InterPro DOMAIN/s: Ubiquitin subgroup (InterPro:IPR019956), Ubiquitin (InterPro:IPR000626), Ubiquitin supergroup (InterPro:IPR019955), HECT (InterPro:IPR000569)	17	301	111	0.15	
AT4G20080	Calcium-dependent lipid-binding (CaLB domain) plant phosphoribosyltransferase family protein; CONTAINS InterPro DOMAIN/s: C2 membrane targeting protein (InterPro:IPR018029), C2 calcium/lipid-binding domain, CaLB (InterPro:IPR008973), Phosphoribosyltransferase C-terminal (InterPro:IPR013583), C2 calcium-dependent membrane targeting (InterPro:IPR000008)	17				
AT4G32915	FUNCTIONS IN: molecular_function unknown; INVOLVED IN: regulation of translational fidelity	17				
AT1G13200	F-box and associated interaction domains-containing protein; FUNCTIONS IN: molecular_function unknown; INVOLVED IN: biological_process unknown	16				
AT1G23490	ADP-ribosylation factor 1 (ARF1); FUNCTIONS IN: protein binding, phospholipase activator activity, GTP binding; INVOLVED IN: response to cadmium ion, N-terminal protein myristoylation	16				
AT1G33780	Protein of unknown function (DUF179); LOCATED IN: chloroplast thylakoid lumen, chloroplast	16				
AT1G34110	Leucine-rich receptor-like protein kinase family protein; FUNCTIONS IN: protein serine/threonine kinase activity, protein kinase activity, ATP binding; INVOLVED IN: transmembrane receptor protein tyrosine kinase signaling pathway, protein amino acid phosphorylation	16	846	385	0.04	
AT2G18700	trehalose phosphatase/synthase 11 (TPS11); FUNCTIONS IN: transferase activity, transferring glycosyl groups; INVOLVED IN: trehalose biosynthetic process, metabolic process	16				
AT2G35500	shikimate kinase like 2 (SKL2); FUNCTIONS IN: shikimate kinase activity, ATP binding; INVOLVED IN: aromatic amino acid family biosynthetic process	16	100	48	0.33	
AT2G38010	Neutral/alkaline non-lysosomal ceramidase; FUNCTIONS IN: ceramidase activity	16				
AT3G28670	oxidoreductase, zinc-binding dehydrogenase family protein; FUNCTIONS IN: oxidoreductase activity, zinc ion binding; INVOLVED IN: oxidation reduction	16				

AT3G30250	like unknown protein [Arabidopsis thaliana] (TAIR:AT1G31150); contains InterPro domain von Willebrand factor, type A; (InterPro:IPR002035)	16				
AT1G08610	Pentatricopeptide repeat (PPR) superfamily protein; CONTAINS InterPro DOMAIN/s: Pentatricopeptide repeat (InterPro:IPR002885)	15	2157	806	0.02	
AT1G52940	purple acid phosphatase 5 (PAP5); CONTAINS InterPro DOMAIN/s: Purple acid phosphatase, N-terminal (InterPro:IPR015914), Metallophosphoesterase (InterPro: IPR004843), Purple acid phosphatase-like, N-terminal (InterPro:IPR008963)	15				
AT2G01650	plant UBX domain-containing protein 2 (PUX2); CONTAINS InterPro DOMAIN/s: PUB domain (InterPro:IPR018997), UBX (InterPro:IPR001012), PUG domain (InterPro:IPR006567)	15				
AT2G01820	Leucine-rich repeat protein kinase family protein; FUNCTIONS IN: protein serine/threonine kinase activity, protein kinase activity, ATP binding; INVOLVED IN: pollen development	15				not detected
AT3G19770	VPS9A; FUNCTIONS IN: Rho guanyl-nucleotide exchange factor activity; INVOLVED IN: cell plate assembly, transport, cell wall biogenesis, post-embryonic root development, embryo development	15				
AT3G25680	unknown protein	15				
AT3G30160	unknown protein	15				
AT4G08250	GRAS family transcription factor; CONTAINS InterPro DOMAIN/s: Transcription factor GRAS (InterPro:IPR005202)	15				
AT4G08450	Disease resistance protein (TIR-NBS-LRR class) family; FUNCTIONS IN: transmembrane receptor activity, nucleoside-triphosphatase activity, nucleotide binding, ATP binding; INVOLVED IN: signal transduction, apoptosis, defense response, innate immune response	15	172	71	0.21	
AT4G11660	AT-HSFB2B; FUNCTIONS IN: transcription repressor activity, sequence-specific DNA binding transcription factor activity; INVOLVED IN: response to chitin	15				
AT5G04370	NAMT1; CONTAINS InterPro DOMAIN/s: SAM dependent carboxyl methyltransferase (InterPro:IPR005299)	15				
