# Supplementary material for: A Genome-Wide DNA Methylation Survey Reveals Salicylic Acid-Induced Distinct Hypomethylation Linked to Defense Responses Against Biotrophic Pathogens
Source: Int J Mol Sci. 2026 Feb 18;27(4):1935. doi: 10.3390/ijms27041935 (PMC12940366; doi:10.3390/ijms27041935)
Supplement: Supplementary file 1 [file ijms-27-01935-s001.zip › Sup_Table_S6.pdf]

Supplementary Table S6. List of identified E3 ubiquitin ligase proteins in the Arabidopsis protein pool that are significantly differentiated between samples. GOBP: Biological process Gene Ontology (GO) term, GOMF: Molecular function GO term, GOCC: Cellular compartment GO term.

Genes	Uniprot function	GOBP name	GOMF name	GOCC name	ANOVA q-value	Protein.Ids	Uniprot full protein name
ABC1K3	Component of the CUL4 RBX1-DDB1-DCAF1 E3 ubiquitin-protein ligase complex, DCAF1 may function as the substrate recognition module within this complex. Appears to be required for plant embryogenesis and to affect several other developmental processes including leaf, shoot, and flower development; Kinase that can phosphorylate the tocopherol cyclaseVTE1, a key enzyme of tocopherol (vitamin E) metabolism and involved in the recycling of oxidated alpha-tocopherol quinone, possibly stabilizing it at plastoglobules. Regulates also membrane prenylquinone composition (PubMed:23632854). Required for photooxidative stress responses to prevent photosystem II core and chlorophyll degradations. Together with ABC1K1, contributes to plastoglobule (PG) function in prenyl-lipid metabolism, stress response, and thylakoid remodeling (PubMed:23673981,PubMed:23632854). Promotes photodamage of chloroplasts under continuous red light, thus working in opposition to ABC1K1(PubMed:25882344).	cellular response to nitrogen starvation; chloroplast organization; embryo development ending in seed dormancy; flower development; fruit development; leaf development; phyllome development; plastoglobule organization; protein stabilization; protein ubiquitination; regulation of tocopherol cyclase activity; response to high light intensity; response to photooxidative stress; response to red light; response to water deprivation; shoot system development	ATP binding; protein kinase activity	chloroplast; Cul4-RING E3 ubiquitin ligase complex; nucleus; plastoglobule	7.41E-05	Q9MA15; Q9M086; Q9MA15	DDB1- and CUL4-associated factor homolog 1; Protein ACTIVITY OF BC1 COMPLEX KINASE 3, chloroplastic {ECO:0000303|PubMed:23673981}
ARI7	Might act as an E3 ubiquitin-protein ligase, or as part of E3 complex, which accepts ubiquitin from specific E2 ubiquitin-conjugating enzymes and then transfers it to substrates	positive regulation of proteasomal ubiquitin-dependent protein catabolic process; protein polyubiquitination; ubiquitin-dependent protein catabolic process	metal ion binding; ubiquitin conjugating enzyme binding; ubiquitin protein ligase activity;	cytoplasm; ubiquitin ligase complex	5.72E-05	Q8L829; Q84RR0; Q8W468	Probable E3 ubiquitin-protein ligase ARI5; Probable E3 ubiquitin-protein ligase ARI7; Probable

			ubiquitin-protein transferase activity				E3 ubiquitin-protein ligase ARI8
At5g58410	E3 ubiquitin-protein ligase. Component of the ribosome quality control complex (RQC), a ribosome-associated complex that mediates ubiquitination and extraction of incompletely synthesized nascent chains for proteasomal degradation. Ubiquitination leads to CDC48 recruitment for extraction and degradation of the incomplete translation product.	proteasome-mediated ubiquitin-dependent protein catabolic process; rescue of stalled ribosome; ribosome-associated ubiquitin-dependent protein catabolic process	ribosomal large subunit binding; ubiquitin protein ligase activity; zinc ion binding	cytosol; RQC complex	8.74E-05	Q9FGI1	E3 ubiquitin-protein ligase listerin
CSU1	RING-finger E3 ubiquitin-protein ligase that plays a major role in maintaining COP1 homeostasis in darkness. Negatively regulates COP1 protein accumulation by targeting COP1 for ubiquitination and subsequent proteasomal degradation in dark-grown seedlings. Negatively regulates the accumulation of SPA1protein in the dark.	photomorphogenesis; protein destabilization	metal ion binding; ubiquitin protein ligase activity	nuclear speck; nucleus	0.001173	Q9SY88	E3 ubiquitin-protein ligase CSU1 {ECO:0000305}
DCAF1	Component of the CUL4-RBX1-DDB1-DCAF1 E3 ubiquitin-protein ligase complex, DCAF1 may function as the substrate recognition module within this complex. Appears to be required for plant embryogenesis and to affect several other developmental processes including leaf, shoot, and flower development; Kinase that can phosphorylate the tocopherol cyclaseVTE1, a key enzyme of tocopherol (vitamin E) metabolism and involved in the recycling of oxidated alpha-tocopherol quinone, possibly stabilizing it at plastoglobules. Regulates also membrane prenylquinone composition (PubMed:23632854). Required for photooxidative stress responses to prevent photosystem II core and chlorophyll degradations. Together with ABC1K1, contributes top lastoglobule	cellular response to nitrogen starvation; chloroplast organization; embryo development ending in seed dormancy; flower development; fruit development; leaf development; phyllome development; plastoglobule organization; protein stabilization; protein ubiquitination; regulation of tocopherol cyclase activity; response to high light intensity; response to photooxidative stress; response to red light; response to water deprivation; shoot system development	ATP binding; protein kinase activity	chloroplast; Cul4-RING E3 ubiquitin ligase complex; nucleus; plastoglobule	0.001199	Q9M086; Q9MA15; Q9M086	DDB1- and CUL4-associated factor homolog 1; Protein ACTIVITY OF BC1 COMPLEX KINASE 3, chloroplastic {ECO:0000303|PubMed:23673981}

	(PG) function in prenyl-lipid metabolism, stress response, and thylakoid remodeling (PubMed:23673981,PubMed:23632854). Promotes photodamage of chloroplasts under continuous red light, thus working in opposition to ABC1K1(PubMed:25882344).						
GRH1	Component of SCF(ASK-cullin-F-box) E3 ubiquitin ligase complexes, which may mediate the ubiquitination and subsequent proteasomal degradation of target proteins. Auxin receptor that mediates Aux/IAA proteins proteasomal degradation and auxin-regulated transcription. Involved in embryogenesis regulation by auxin. Confers sensitivity to the virulent bacterial pathogen P. syringae. Mediates glucose repression in yeast	auxin-activated signaling pathway; carbon catabolite repression of transcription by glucose; defense response; pollen maturation; response to auxin; SCF-dependent proteasomal ubiquitin-dependent protein catabolic process	auxin binding; inositol hexakisphosphate binding; ubiquitin-protein transferase activity	nuclear SCF ubiquitin ligase complex; nucleus; SCF ubiquitin ligase complex	0.002462	Q9ZR12	GRR1-like protein 1
HRD3A	Component of the endoplasmic reticulum (ER) quality control system called ER-associated degradation (ERAD) and involved in ubiquitin-dependent degradation of misfolded endoplasmic reticulum proteins. Functions as an ERAD substrate-recruiting factor that recognizes misfolded proteins for the HRD1E3 ubiquitin ligase complex. Targets the misfolded LRR receptor kinase BRI1.	hyperosmotic salinity response; ubiquitin-dependent ERAD pathway		endoplasmic reticulum; endoplasmic reticulum membrane; integral component of membrane; membrane	0.000191	Q9LM25	ERAD-associated E3 ubiquitin-protein ligase component HRD3A {ECO:0000305}
HSP90-1	Functions as a holding molecular chaperone (holdase)which stabilizes unfolding protein intermediates and rapidly releases them in an active form once stress has abated. Functions as a folding molecular chaperone (foldase) that assists the non-covalent folding of proteins in an ATP-dependent manner (PubMed:23827697). Molecular chaperone involved in R gene-mediated disease resistance. Required for full RPS2-mediated resistance through interaction with RAR1. Possesses	cellular response to calcium ion; cellular response to heat; chaperone-mediated protein complex assembly; chaperone-mediated protein folding; defense response to bacterium, incompatible interaction; Innate immune response; protein folding; protein stabilization; response to heat	ATP binding; ATPase activity; unfolded protein binding	apoplast; cell surface; cell wall; chloroplast stroma; cytoplasm; cytosol; Golgi apparatus; membrane; nucleolus; nucleus; perinuclear region of cytoplasm; plasma membrane; protein-containing complex; vacuolar membrane	0.000111	P27323; O03986; P55737; P51818; P27323; O03986; P55737; P51818; P27323	Heat shock protein 90-1 {ECO:0000305}; Heat shock protein 90-2 {ECO:0000305}; Heat shock protein 90-3 {ECO:0000305}; Heat shock protein 90-4 {ECO:0000305}

	probably ATPase activity (PubMed:14504384).; Functions as a holding molecular chaperone (holdase)which stabilizes unfolding protein intermediates and rapidly releases them in an active form once stress has abated. Functions as a folding molecular chaperone (foldase) that assists the non-covalent folding of proteins in an ATP-dependent manner (PubMed:23827697). Regulates RPP4-mediated temperature-dependent cell death and defense responses (PubMed:24611624). May assist SGT1B in the formation of SCF E3 ubiquitin ligase complexes that target the immune receptors SNC1, RPS2 and RPS4 for degradation, to regulate receptor levels and avoid autoimmunity(PubMed:24889324).; Molecular chaperone which stabilizes unfolding protein intermediates and functions as a folding molecular chaperone that assists the non-covalent folding of proteins in an ATP-dependent manner.; Molecular chaperone. Involved in RPM1-mediatedresistance. Component of the RPM1/RAR1/SGT1 complex. May stabilizeRPM1 and protect it from SGT1-mediated degradation. Associates with RAR1 which may function as co-chaperone. Possesses ATPase activity (PubMed:14592967, PubMed:19487680). In the absence of heat shock, negatively regulates heat-inducible genes by actively suppressing heat shock transcription factor A1D (HSFA1D) function(PubMed:17965410). Involved in the induction of heat shock transcription factor A2 (HSFA2) expression in response to oxidative stress (PubMed:20147301). Required for stomatal closure and modulates						

	transcriptional and physiological responses to abscisic acid (ABA) (PubMed:21586649). Regulates RPP4-mediatedtemperature-dependent cell death and defense responses (PubMed:24611624). May assist SGT1B in the formation of SCF E3ubiquitin ligase complexes that target the immune receptors SNC1,RPS2 and RPS4 for degradation, to regulate receptor levels and avoid autoimmunity (PubMed:24889324)						
NBR1	Autophagic substrate degraded in the vacuole by non-selective autophagy. Requires ATG8 protein expression to be recognized as an autophagic substrate (PubMed:21606687). Acts probably as a receptor for autophagosomal degradation of ubiquitinated proteins. Targets ubiquitinated protein aggregates derived from denatured or damaged non-native proteins generated under stress conditions (PubMed:23341779). Functions additively with the E3 ubiquitin-protein ligase CHIP for autophagosomal degradation of proteotoxic aggregates formed under stress conditions (PubMed:24497840).	macroautophagy; protein polymerization; protein transport	ubiquitin binding; zinc ion binding	cytoplasm; vacuole	1.36E-05	Q9SB64	Protein NBR1 homolog {ECO:0000305}
PAD1	The proteasome is a multi catalytic proteinase complex which is characterized by its ability to cleave peptides with Arg,Phe, Tyr, Leu, and Glu adjacent to the leaving group at neutral orslightly basic pH. The proteasome has an ATP-dependent proteolyticactivity.; The proteasome is a multi catalytic proteinase complex which is characterized by its ability to cleave peptides with Arg, Phe, Tyr, Leu, and Glu adjacent to the leaving group at neutral or slightly basic pH. The proteasome has an ATP-dependent proteolytic activity. Mediates the association of the SCF (TIR1) E3	proteasomal protein catabolic process; proteasomal ubiquitin-independent protein catabolic process; proteasome-mediated ubiquitin-dependent protein catabolic process; ubiquitin-dependent protein catabolic process	endopeptidase activity; threonine-type endopeptidase activity	chloroplast; cytoplasm; cytosol; cytosolic ribosome; nucleus; phragmoplast; proteasome complex; proteasome core complex; proteasome core complex, alpha-subunit complex; spindle; vacuolar membrane; vacuole	5.68E-05	O24616; P30186; P30186	Proteasome subunit alpha type-7-A; Proteasome subunit alpha type-7-B

	ubiquitin ligase complex with the proteasome.						
PAD2	The proteasome is a multi catalytic proteinase complex which is characterized by its ability to cleave peptides with Arg, Phe, Tyr, Leu, and Glu adjacent to the leaving group at neutral or slightly basic pH. The proteasome has an ATP-dependent proteolytic activity.; The proteasome is a multi catalytic proteinase complex which is characterized by its ability to cleave peptides with Arg, Phe, Tyr, Leu, and Glu adjacent to the leaving group at neutral or slightly basic pH. The proteasome has an ATP-dependent proteolytic activity. Mediates the association of the SCF(TIR1) E3 ubiquitin ligase complex with the proteasome.	proteasomal protein catabolic process; proteasomal ubiquitin-independent protein catabolic process; proteasome-mediated ubiquitin-dependent protein catabolic process; ubiquitin-dependent protein catabolic process	endopeptidase activity; threonine-type endopeptidase activity	chloroplast; cytoplasm; cytosol; cytosolic ribosome; nucleus; phragmoplast; proteasome complex; proteasome core complex; proteasome core complex, alpha-subunit complex; spindle; vacuolar membrane; vacuole	0	O24616; O24616; P30186	Proteasome subunit alpha type-7-A; Proteasome subunit alpha type-7-B
PUB4	Functions as an E3 ubiquitin ligase.		ubiquitin-protein transferase activity	cytoplasm; nucleus	0.002798	O22193	U-box domain-containing protein 4
RGLG4	Possesses E3 ubiquitin-protein ligase in vitro. Acts as upstream modulator of jasmonate (JA) signaling in response to various stimuli, such as JA-inhibited root growth, JA-inductive gene expression, coronatine-mediated pathogen susceptibility, wound-stimulated expression of JA-responsive genes and wound-induced JA biosynthesis (PubMed:22898498). Controls fumonisin B1(FB1)-triggered programmed cell death (PCD) by modulating the JA signaling pathway. May mediate salicylic acid (SA) suppression of JA signaling in FB1-induced responses (PubMed:25788731). May mediate the formation of 'Lys-48'-linked multiubiquitin chains. Mediates the polyubiquitination and subsequent proteasomal degradation of the target protein GRXS17 (PubMed:27497447)	defense response to bacterium; jasmonic acid mediated signaling pathway; response to wounding	metal ion binding; ubiquitin-protein transferase activity	cytoplasm; nucleus	0.000393	Q9SAL0	E3 ubiquitin-protein ligase RGLG4 {ECO:0000305}

SKP1A	Involved in ubiquitination and subsequent proteasomal degradation of target proteins. Together with CUL1, RBX1 and a F-box protein, it forms a SCF E3 ubiquitin ligase complex. The functional specificity of this complex depends on the type of F-box protein. In the SCF complex, it serves as an adapter that links the F-box protein to CUL1. SCF(UFO) is required for vegetative and floral organ development as well as for male gametogenesis. SCF(TIR1) is involved in auxin signaling pathway. SCF(COI1) regulates responses to jasmonates. SCF(EID1) and SCF(AFR) are implicated in phytochrome A light signaling. SCF(ADO1), SCF(ADO2), SCF(ADO3) are related to the circadian clock. SCF(ORE9) seems to be involved in senescence. SCF(EBF1/EBF2) may regulate ethylene signaling. Plays a role during embryogenesis and early postembryonic development, especially during cell elongation and division. Contributes to the correct chromosome segregation during tetrad formation	auxin-activated signaling pathway; chromosome segregation; embryo development ending in seed dormancy; ethylene-activated signaling pathway; microtubule cytoskeleton organization; multicellular organism development; negative regulation of DNA recombination; protein ubiquitination; response to cadmium ion; ubiquitin-dependent protein catabolic process; viral process		cytosol; nucleus; phragmoplast; plasma membrane; spindle	0.000146	Q39255; Q39255; Q9FHW7	SKP1-like protein 1A {ECO:0000303|PubMed:10778750}; SKP1-like protein 1B
SR1IP1	Acts as a substrate-specific adapter of an E3 ubiquitin-protein ligase complex (CUL3-RBX1-BTB) which mediates the ubiquitination and subsequent proteasomal degradation of target proteins (Probable). Involved in disease resistance. Acts as a substrate adapter that recruits CAMTA3/SR1 for ubiquitination and degradation during pathogen infection. Acts as positive regulator of plant defense by removing the defense suppressor CAMTA3/SR1(PubMed:24528504).	defense response to bacterium; protein ubiquitination			1.70E-05	Q66GP0	BTB/POZ domain-containing protein SR1IP1 {ECO:0000305}
VIP1	Bifunctional inositol kinase that acts in concert with the IP6K kinases to synthesize the diphosphate group-	cellular response to sulfate starvation; defense response; defense response to fungus, incompatible interaction; DNA	5-diphosphoinositol pentakisphosphate 3-kinase activity; ATP	cytosol; nucleus; plasma membrane	0.000786	F4J8C6; F4J8C6;	Inositol hexakisphosphate and diphosphoinositol-

	containing inositol pyrophosphates diphosphoinositol pentakisphosphate, PP-InsP5, and bis-diphosphoinositol tetrakisphosphate, (PP)2-InsP4.PP-InsP5 and (PP)2-InsP4, also respectively called InsP7 andInsP8, may regulate a variety of cellular processes, including apoptosis, vesicle trafficking, cytoskeletal dynamics, and exocytosis. Phosphorylates inositol hexakisphosphate (InsP6) at positions 1 or 3 to produce PP-InsP5 which is in turn phosphorylated by IP6Ks to produce (PP)2-InsP4. Alternatively, phosphorylates at position 1 or 3 PP-InsP5, produced by IP6Ks fromInsP6, to produce (PP)2-InsP4 (PubMed:25231822). Probably involved in vitamin E homeostasis via the regulation of gamma-to copherol biosynthesis (By similarity).; Bifunctional inositol kinase that acts in concert with the IP6K kinases to synthesize the diphosphate group-containing inositol pyrophosphates diphosphoinositol pentakisphosphate, PP-InsP5, and bis-diphosphoinositol tetrakisphosphate, (PP)2-InsP4.PP-InsP5 and (PP)2-InsP4, also respectively called InsP7 andInsP8, may regulate a variety of cellular processes, including apoptosis, vesicle trafficking, cytoskeletal dynamics, andexocytosis. Phosphorylates inositol hexakisphosphate (InsP6) at positions 1 or 3 to produce PP-InsP5 which is in turn phosphorylated by IP6Ks to produce (PP)2-InsP4. Alternatively, phosphorylates at position 1 or 3 PP-InsP5, produced by IP6Ks fromInsP6, to produce (PP)2-InsP4 (PubMed:25231822). Probably involved in vitamin E homeostasis via the regulation of gamma-to copherol biosynthesis (PubMed:17077148).	mediated transformation; import into nucleus; inositol metabolic process; inositol phosphate biosynthetic process; jasmonic acid and ethylene-dependent systemic resistance; negative regulation of cell differentiation; osmosensory signaling pathway; positive regulation of antifungal innate immune response; positive regulation of defense response to insect; positive regulation of vitamin E biosynthetic process; response to osmotic stress; sulfate transport; thigmotropism	binding; chromatin binding; diphosphoinositol-pentakisphosphate kinase activity; DNA-binding transcription factor activity; inositol heptakisphosphate kinase activity; inositol hexakisphosphate 1-kinase activity; inositol hexakisphosphate 3-kinase activity; inositol hexakisphosphate 5-kinase activity; inositol hexakisphosphate kinase activity; mitogen-activated protein kinase binding; nucleic acid binding; protein self-association; sequence-specific DNA binding			Q84WW3; Q9MA75	pentakisphosphate kinase VIP1 {ECO:0000303|PubMed:25231822}; Inositol hexakisphosphate and diphosphoinositol-pentakisphosphate kinase VIP2 {ECO:0000303|PubMed:25231822}; Transcription factor VIP1

	Catalyzes the conversion of InsP7to InsP8. Regulates jasmonic acid (JA) perception and plant defenses against herbivorous insects (e.g. P.rapae) and necrotrophic fungi (e.g. M.brassicae, B.cinerea and A.brassicicola) by triggering the production of jasmonate-induced pools of InsP8 and subsequent activation of SCF(COI1) E3 ubiquitin ligase complexes with JAZ proteins (e.g. TIFY10A/JAZ1)(PubMed:25901085).; Transcription activator that binds specifically to theVIP1 response elements (VREs) DNA sequence 5'-ACNGCT-3' found insome stress genes (e.g. TRX8 and MYB44), when phosphorylated/activated by MPK3. Required for Agrobacterium VirE2nuclear import and tumorigenicity. Promotes transient expression of T-DNA in early stages by interacting with VirE2 in complex with the T-DNA and facilitating its translocation to the nucleus, and mediates stable genetic transformation by Agrobacterium by bindingH2A histone. Prevents cell differentiation and shoot formation. Limits sulfate utilization efficiency (SUE) and sulfate uptake, especially in low-sulfur conditions.						
