# Supplementary material for: A Genome-Wide DNA Methylation Survey Reveals Salicylic Acid-Induced Distinct Hypomethylation Linked to Defense Responses Against Biotrophic Pathogens
Source: Int J Mol Sci. 2026 Feb 18;27(4):1935. doi: 10.3390/ijms27041935 (PMC12940366; doi:10.3390/ijms27041935)
Supplement: Supplementary file 1 [file ijms-27-01935-s001.zip › Sup_Table_S7.pdf]

Supplementary Table S7. List of genes common in hypomethylated DMCs, in the SA-CNPs vs control comparison (>5 DMCs and >25% differential hypomethylation), and in upregulated differentially expressed genes (DEGs) identified in this comparison.

Gene	No of DMCs	FoldChange	Gene name	Description
AT1G35230	40	5.117293568	AGP5	Proteoglycan that seems to be implicated in diverse developmental roles such as differentiation, cell-cell recognition, embryogenesis and programmed cell death
AT1G66700	13	3.7717211	PXMT1	Methyltransferase that may methylate 1,7-paraxanthine
AT4G04540	13	8.712108975	CRK39	Putative cysteine-rich receptor-like protein kinase 39
AT3G04070	11	3.439269096	NAC047	Transcription factor that binds to the promoter of ACO5
AT3G60961	11	30.42301015		P-loop containing nucleoside triphosphate hydrolases superfamily
AT2G35980	10	6.183029976	NHL10	NDR1/HIN1-like protein 10
AT1G08630	8	2.654343348	THA1	Low-specificity L-threonine aldolase 1
AT1G51800	8	4.204327343	IOS1	LRR receptor-like serine/threonine-protein kinase IOS1
AT2G05520	8	2.442725922	GRP3	Glycine-rich protein 3; Regulates the function of the receptor protein kinase WAK1, and namely the phosphorylation of OEE2
AT2G16910	8	7.781174857	AMS	Transcription factor ABORTED MICROSPORES
AT3G28580	7	6.377256902		AAA-ATPase At3g28580
AT4G08770	7	7.978944991	PER37	Peroxidase 37
AT4G21400	7	2.001249441	CRK28	Cysteine-rich receptor-like protein kinase 28
AT5G56960	7	15.13668103	BHLH41	Putative transcription factor bHLH041
AT1G13470	6	12.72025776		Uncharacterized protein At1g13470
AT1G58370	6	3.132516264	XYN1	Endo-1,4-beta-xylanase 1
AT1G58430	6	6.578095713		GDSL esterase/lipase At1g58430
AT1G72260	6	71.77323286	THI2.1	Thionin-2.1
AT2G04160	6	2.669608157	AIR3	Subtilisin-like protease SBT5.3
AT3G27380	6	2.104598452	SDH2-1	Succinate dehydrogenase [ubiquinone] iron-sulfur subunit 1, mitochondrial

AT3G57460	6	7.636698147		Catalytic/ metal ion binding / metalloendopeptidase/ zinc ion binding protein
AT4G39890	6	2.816184785	RABH1C	Ras-related protein RABH1c
AT5G25440	6	3.184590784	SZE1	Serine/threonine-protein kinase SZE1
AT5G36925	6	2.162406869	ARACIN1	Peptide ARACIN 1
AT1G17590	5	2.36881577	NFYA8	Nuclear transcription factor Y subunit A-8
AT1G51620	5	4.631299316		
AT1G52040	5	2.149419381	MBP1	Myrosinase-binding protein 1
AT1G61420	5	2.885437336		G-type lectin S-receptor-like serine/threonine-protein kinase At1g61420
AT2G29110	5	4.026560471	GLR2.8	Glutamate receptor 2.8
AT3G11840	5	2.609170098	PUB24	E3 ubiquitin-protein ligase PUB24
AT3G17700	5	2.031158748	CNGC20	Probable cyclic nucleotide-gated ion channel 20, chloroplastic
AT3G20410	5	2.114220722	CPK9	Calcium-dependent protein kinase 9
AT3G49160	5	7.849844089	PKP4	Plastidial pyruvate kinase 4, chloroplastic
AT3G61280	5	6.706857734		Glycosyltransferase
AT4G11521	5	2.218877467		Putative cysteine-rich receptor-like protein kinase At4g11521
AT4G38560	5	3.197488122		phospholipase-like protein (PEARLI 4) family protein
AT4G39030	5	4.839379236	DTX7	Protein DETOXIFICATION 47, chloroplastic (EDS5)
AT5G04930	5	2.105615479	ALA1	Phospholipid-transporting ATPase 1
AT5G24540	5	81.40220674	BGLU31	Beta-glucosidase 31
AT5G33290	5	2.391488742	XGD1	Xylogalacturonan beta-1,3-xylosyltransferase
