# Supplementary figures and images for: A Genome-Wide DNA Methylation Survey Reveals Salicylic Acid-Induced Distinct Hypomethylation Linked to Defense Responses Against Biotrophic Pathogens
Source: Int J Mol Sci. 2026 Feb 18;27(4):1935. doi: 10.3390/ijms27041935 (PMC12940366; doi:10.3390/ijms27041935)

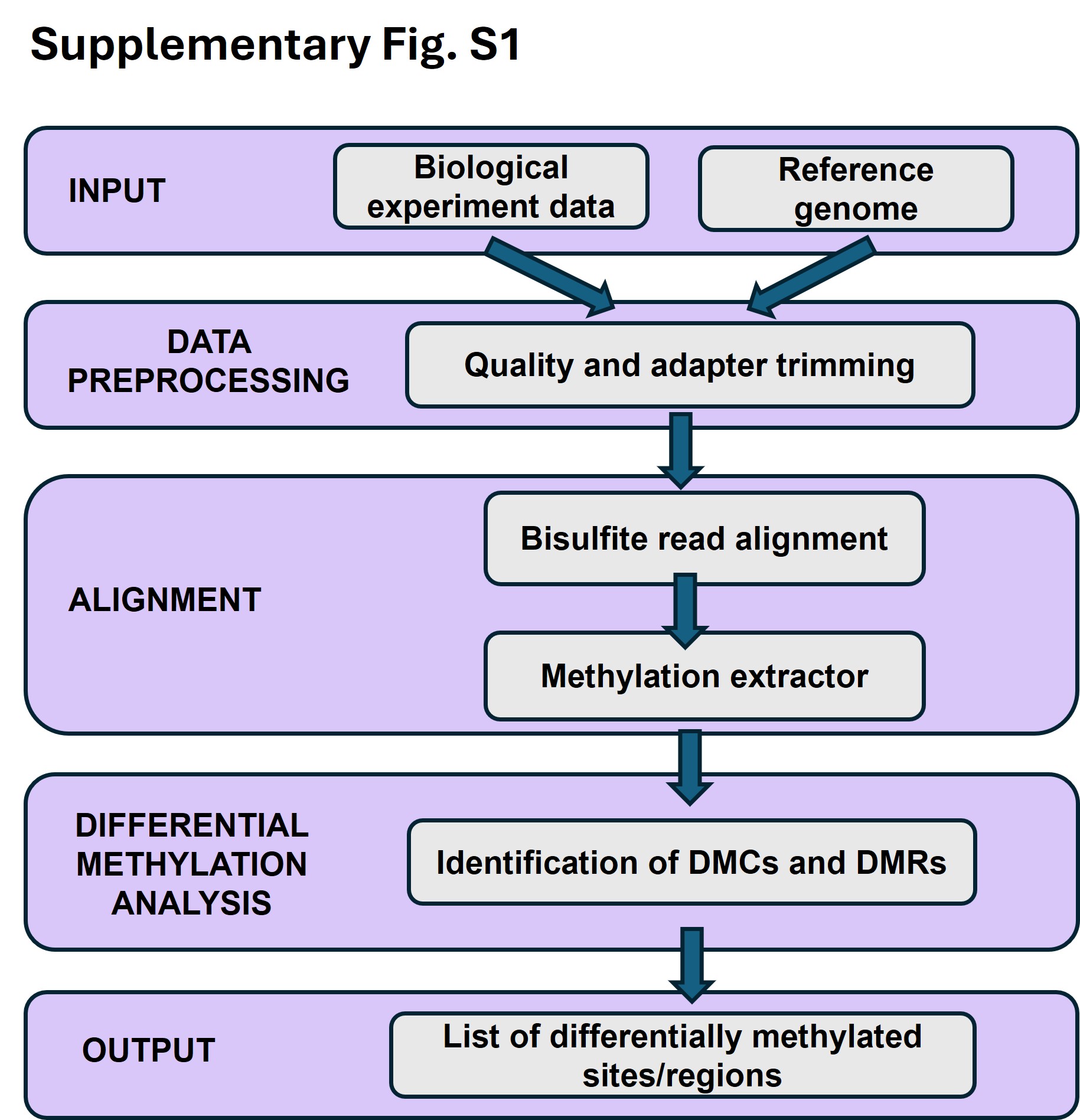

Supplement: Supplementary file 1 [file ijms-27-01935-s001.zip › Sup_Fig_S1.jpg]

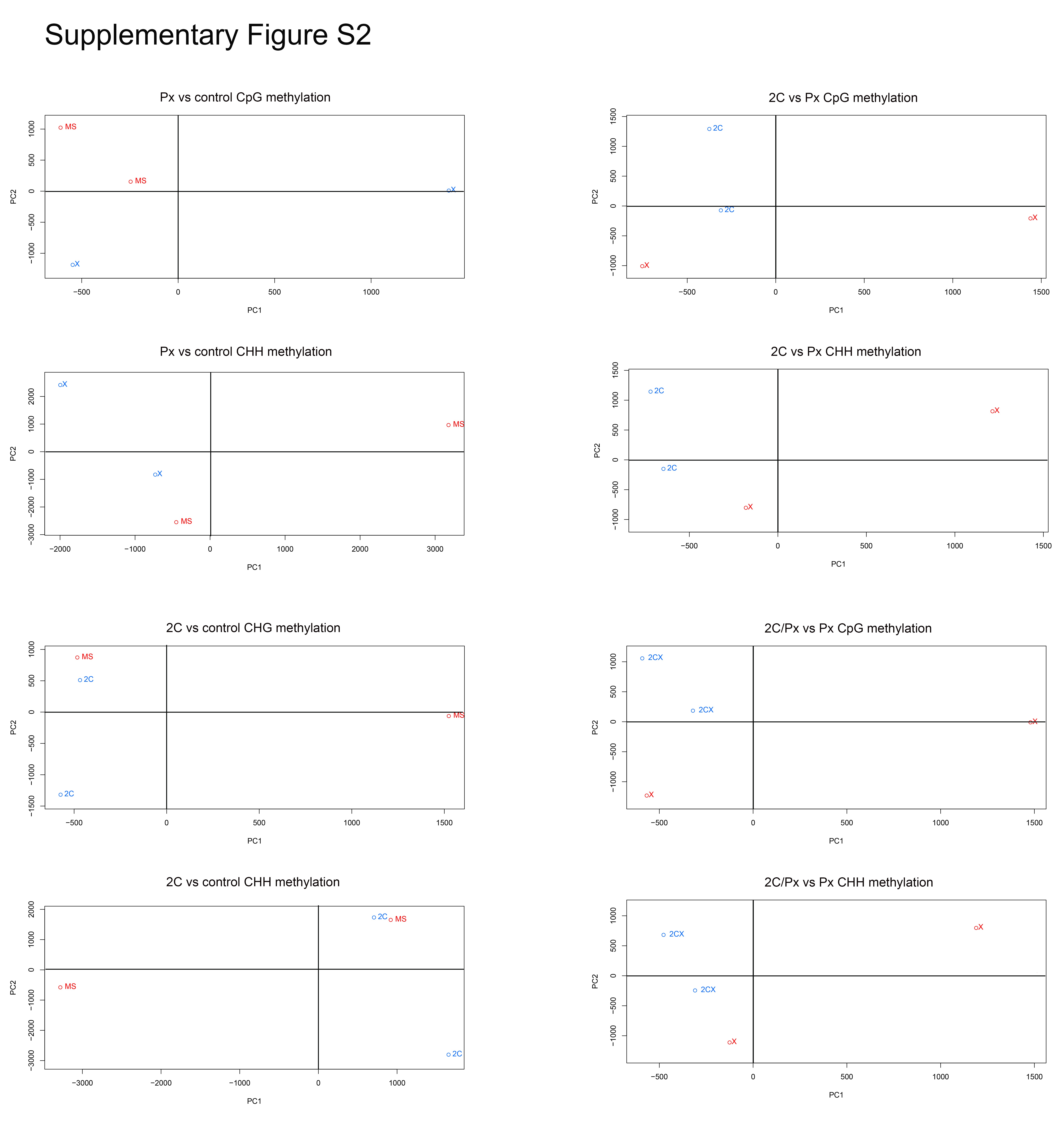

Supplement: Supplementary file 1 [file ijms-27-01935-s001.zip › Sup_Fig_S2.jpg]

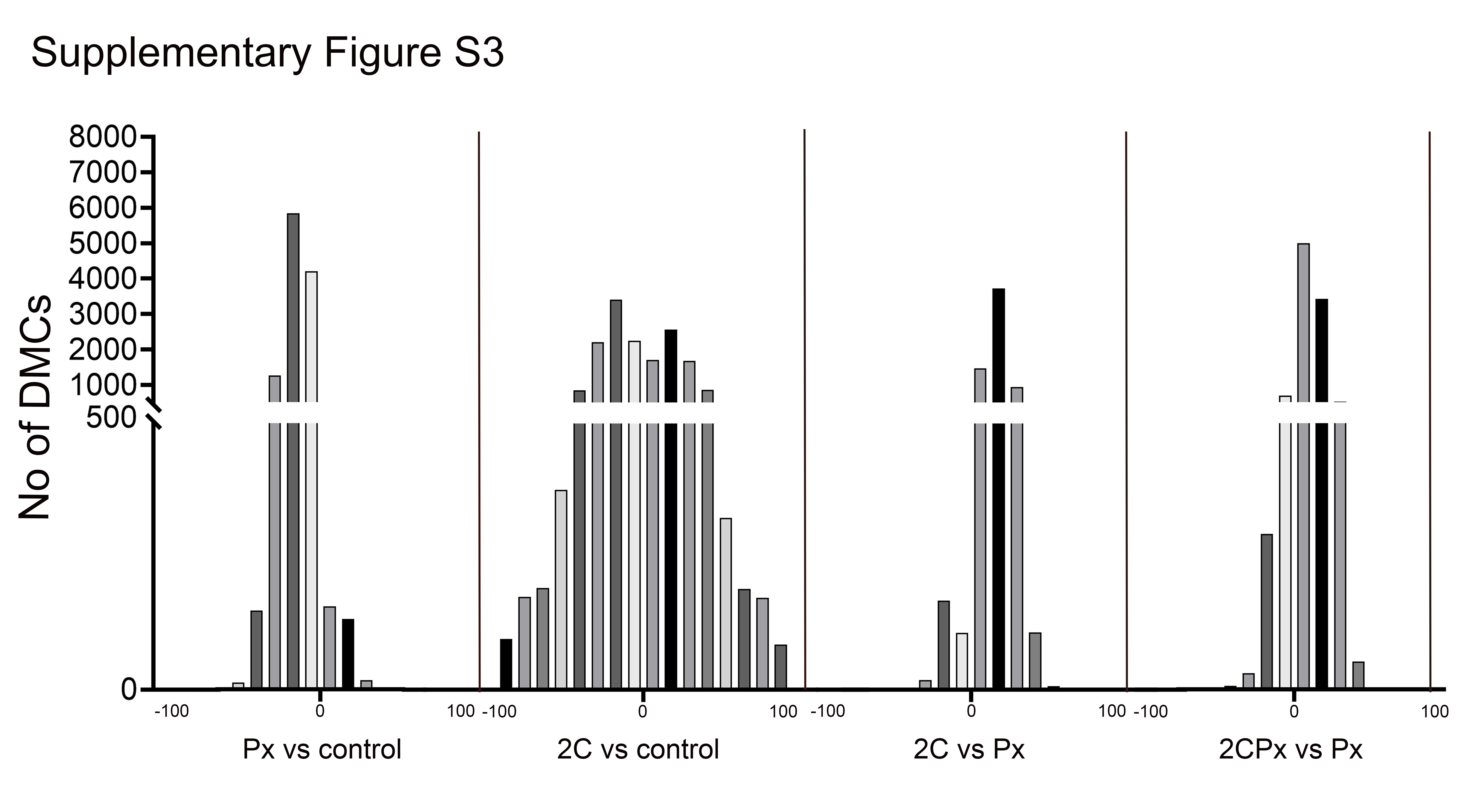

Supplement: Supplementary file 1 [file ijms-27-01935-s001.zip › Sup_Fig_S3.jpg]

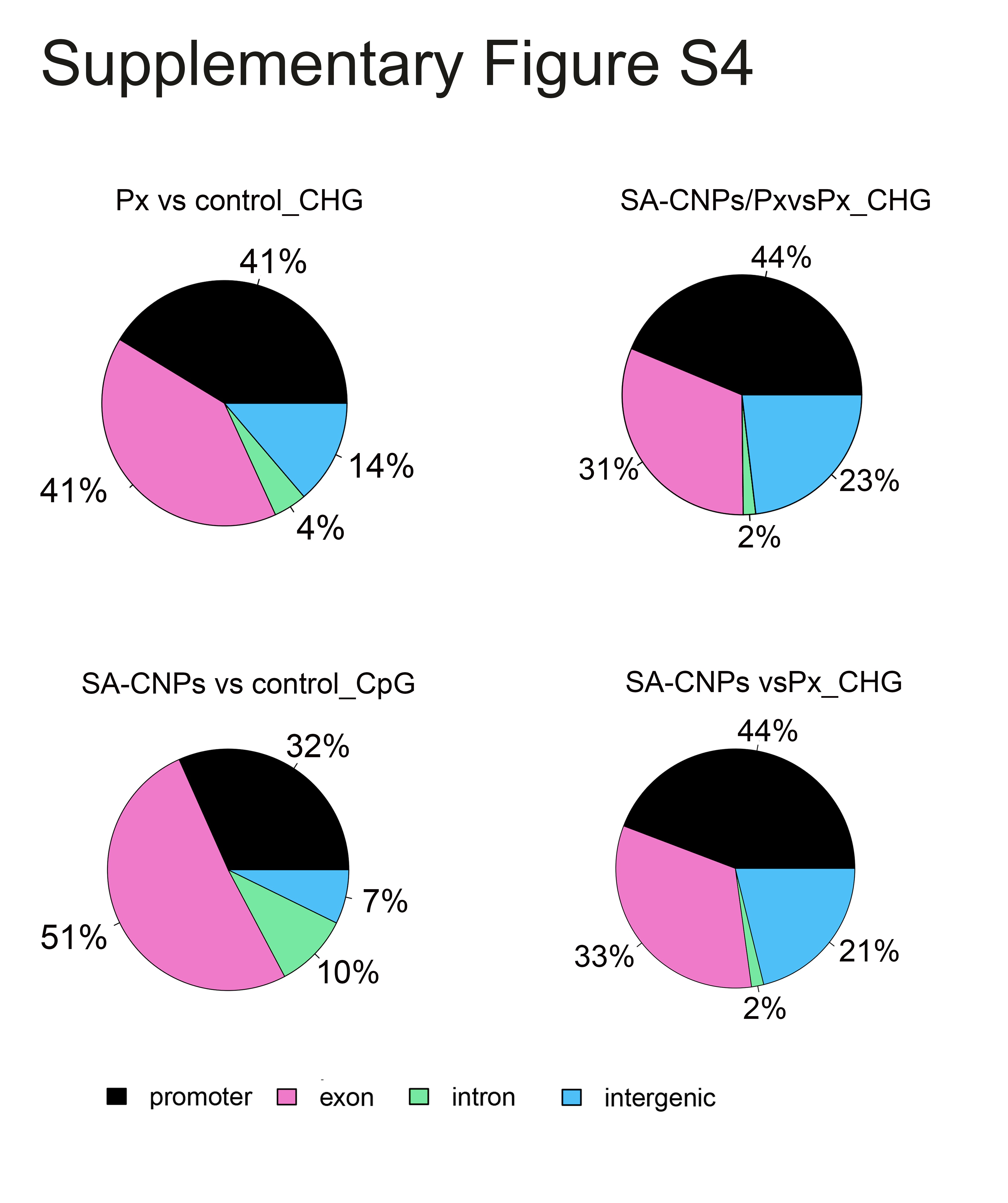

Supplement: Supplementary file 1 [file ijms-27-01935-s001.zip › Sup_Fig_S4.jpg]

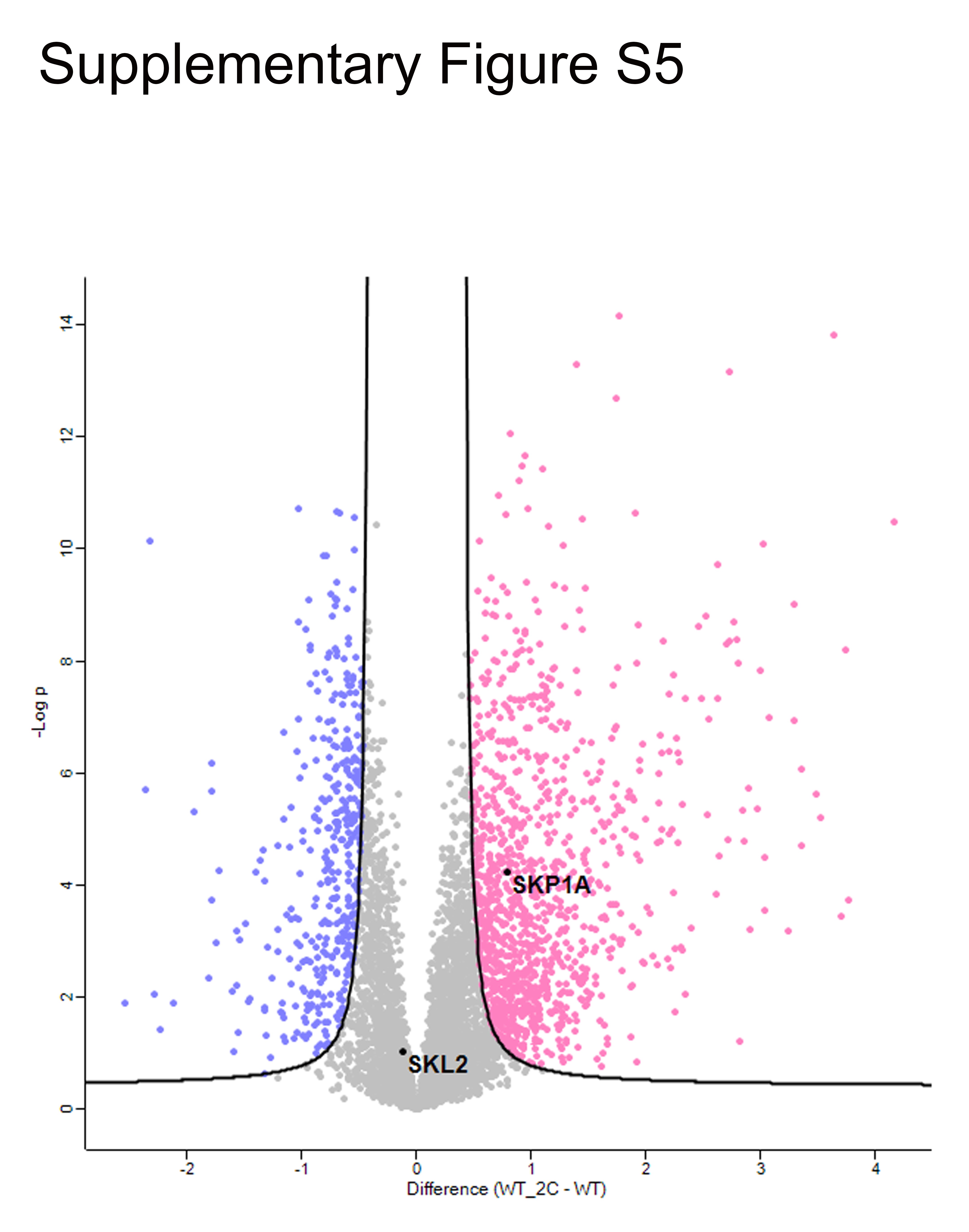

Supplement: Supplementary file 1 [file ijms-27-01935-s001.zip › Sup_Fig_S5.jpg]

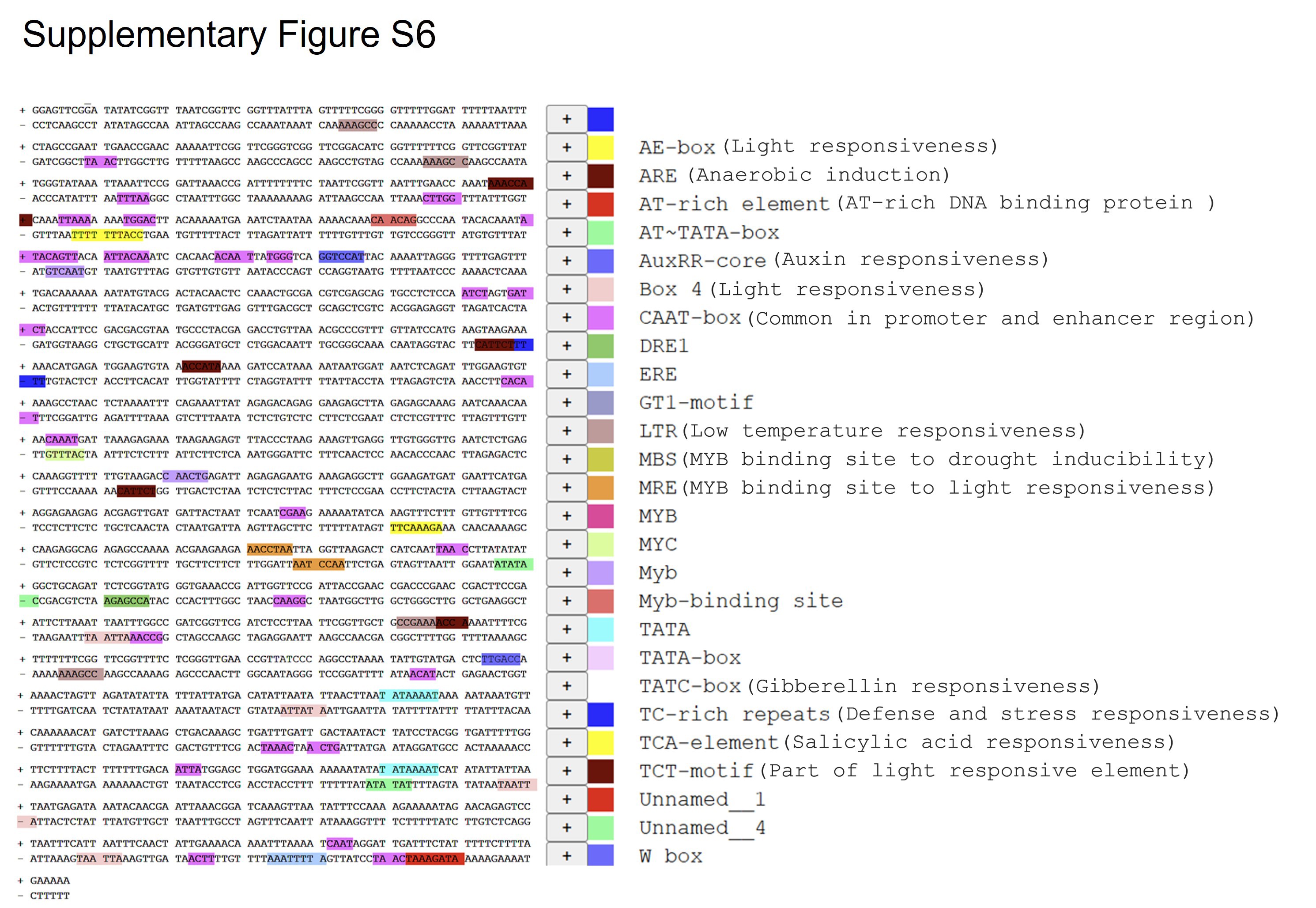

Supplement: Supplementary file 1 [file ijms-27-01935-s001.zip › Sup_Fig_S6.jpg]

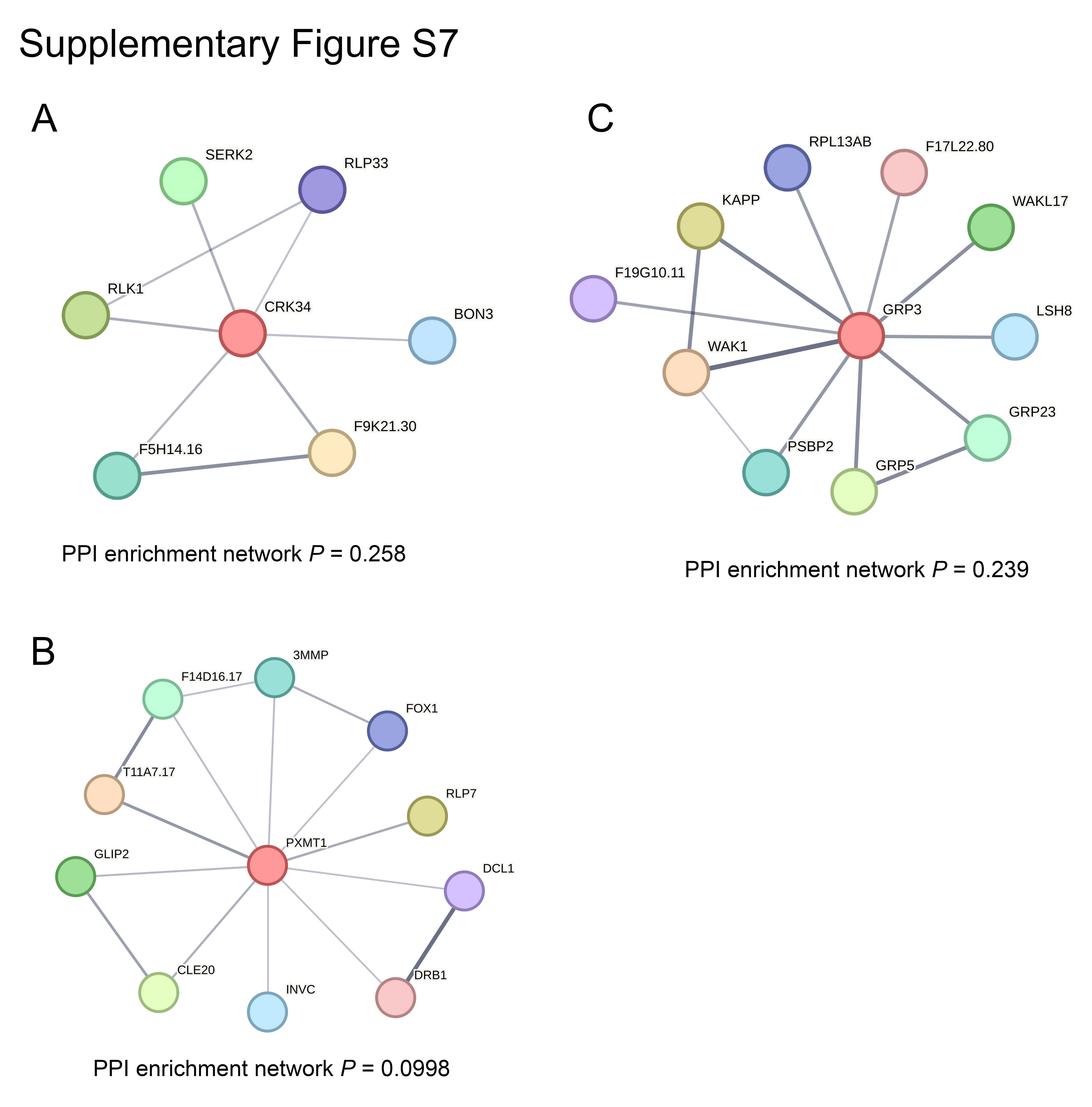

Supplement: Supplementary file 1 [file ijms-27-01935-s001.zip › Sup_Fig_S7.jpg]
